# The effect of sertraline on networks of mood and anxiety symptoms: secondary analysis of the PANDA randomized controlled trial

**DOI:** 10.1038/s44220-025-00528-x

**Published:** 2025-10-30

**Authors:** Giulia G. Piazza, Andrea G. Allegrini, Larisa Duffy, Gemma Lewis, Glyn Lewis, Jonathan P. Roiser, Jean-Baptiste Pingault

**Affiliations:** 1 Department of Clinical, Educational and Health Psychology, University College London, London, UK; 2 Social, Genetic and Developmental Psychiatry Centre, King’s College London, London, UK; 3 Division of Psychiatry, University College London, London, UK; 4 Institute of Cognitive Neuroscience, University College London, London, UK

**Keywords:** Depression, Psychology

## Abstract

Depression consists of heterogeneous symptoms that can occur in hundreds of possible combinations. However, intervention studies commonly operationalize depression as a homogeneous condition. Here we adopt a symptom-level approach to test the effects of the selective serotonin reuptake inhibitor sertraline on depression and anxiety symptoms and to test their associations. Using data from the PANDA randomized controlled trial, we use network models to estimate the effects of sertraline at different time points (contemporaneous networks at 2, 6 and 12 weeks) and across time (temporally lagged networks). Results show that sertraline has beneficial effects on core depression and anxiety symptoms as early as after 2 weeks of treatment, counteracted by detrimental effects on somatic symptoms of depression. This intricate pattern of treatment effects is typically masked when measuring depression on a single dimension. Focusing on individual symptoms of depression and anxiety may shed light on the nature, effectiveness and timing of antidepressant action.

## Abstract (web summary)

This research adopts a symptom-level approach to evaluate the effects of sertraline on depression and anxiety using network models from the PANDA trial, revealing early benefits for core symptoms while highlighting adverse impacts on somatic manifestations over time.

## Main

Selective serotonin reuptake inhibitors (SSRIs) are a first-line treatment for depression and anxiety. Although meta-analytic evidence suggests that they have modest effect sizes compared with placebo^1,2^, SSRIs have been increasingly prescribed in recent years^3^. The response to antidepressants can take weeks to develop, and relatively little is known about the precise mechanism of action behind it^4,5^.

Multiple lines of evidence indicate considerable heterogeneity in symptoms of depression and anxiety. For example, some symptoms of depression, such as sad mood and concentration problems, show larger associations with functional impairment compared with other symptoms, such as weight and appetite problems^6^. Symptoms of depression are also differentially associated with environmental and genetic risk factors^7,8^; for instance, appetite changes and fatigue appear to have higher heritability estimates^7^. Isolation and grief have been associated with crying and sadness, while chronic stress is associated with fatigue and hypersomnia^9^.

Similarly, studies focusing on individual symptoms have reported differential treatment responses to SSRIs across symptom subgroups^10–14^. Commonly used SSRIs were found to be more effective at treating core emotional symptoms than somatic symptoms^15^, suggesting that they may simultaneously be effective in alleviating a subset of symptoms while failing to treat or even exacerbating others.

In addition, reciprocal causal associations between symptoms may lead to maladaptive cycles^16^. For example, insomnia might cause concentration problems, which could, in turn, reduce self-esteem. Separating the direct and indirect effects of SSRIs on individual symptoms has potentially important implications for understanding the mechanisms underlying interventions^17^.

Network analysis is a useful framework that allows for the statistical modeling and visualization of symptoms and their associations^18^. In networks, symptoms are represented by nodes, while their associations are represented as edges between nodes^19^. In this framework, SSRIs could exert direct effects on individual symptoms, for example, by directly improving mood. In addition, network analysis can examine network structures, that is, the presence or absence and magnitude of associations between symptoms. SSRIs could alter network structures^20^, for example, by reducing the strength of the association between feelings of sadness and feelings of guilt.

Network studies have suggested that antidepressant treatment is associated with improvements in individual symptoms of depression and anxiety, such as feelings of guilt^21^, anxiety and avoidance^22,23^, depressed mood^24^ and worry^25^. However, few such studies have included a placebo group^22,24^, which precludes drawing strong conclusions, and most have only compared pre- and posttreatment networks cross-sectionally^17,23,25–28^, neglecting potentially important temporal associations between symptoms. New insights into the effects of sertraline can emerge from modeling temporal associations between symptoms in both treatment and placebo groups.

Therefore, this study tests the direct effects of SSRI treatment on symptoms of depression and anxiety, relative to placebo, both at a single time point and across time and examines associations between these symptoms. Combining analytical approaches, we conduct a secondary analysis of a large placebo-controlled randomized trial on the effectiveness of sertraline for the treatment of depression (the PANDA trial^29^). First, using a standard regression approach, we investigate the effects of sertraline on individual depression and anxiety symptoms, compared with placebo. Second, we investigate these effects while accounting for associations between symptoms with network analyses, at each time point (contemporaneous networks) and across time (temporally lagged networks). Third, we compare the patterns of associations between symptoms (that is, network structures, both contemporaneously and across time) between sertraline and placebo groups. On the basis of the primary results of the PANDA trial (using sum-scores), we predicted a beneficial effect of sertraline on depression symptoms by 12 weeks of treatment, compared with placebo. At the symptom level, drawing on existing literature^17,23,24,28,30–33^, we anticipated direct beneficial effects of sertraline on depressed mood and worry, relative to placebo, when accounting for associations with all other symptoms. We expected these effects to be detectable both in contemporaneous and temporally lagged symptom networks, with changes emerging by 12 weeks of treatment.

## Results

### Effect of sertraline on individual symptoms

**Fig. 1:**
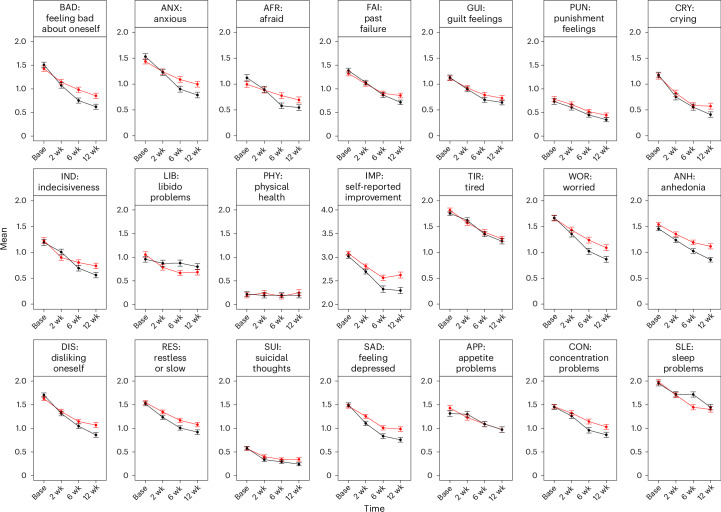
Effects of treatment and time on mean symptoms. Means ( ± standard errors) of symptoms of depression and anxiety (derived in the node selection step) at baseline, 2 weeks, 6 weeks and 12 weeks (*n*_max _= 571). AFR, feeling afraid; ANH, loss of interest and pleasure in everyday life; ANX, feeling nervous or anxious; APP, lack of appetite or eating too much; BAD, feeling bad about oneself; CON, concentration problems; CRY, crying; DIS, disliking oneself; FAI, past failure; GUI, guilt feelings; IMP, self-reported improvement; IND, indecisiveness; LIB, loss of interest in sex; PHY, general physical health; PUN, punishment feelings; RES, being restless or slow; SAD, feeling sad or depressed; SLE, sleep problems; SUI, suicidal thoughts; TIR, feeling tired; WOR, feeling worried.

**Fig. 2:**
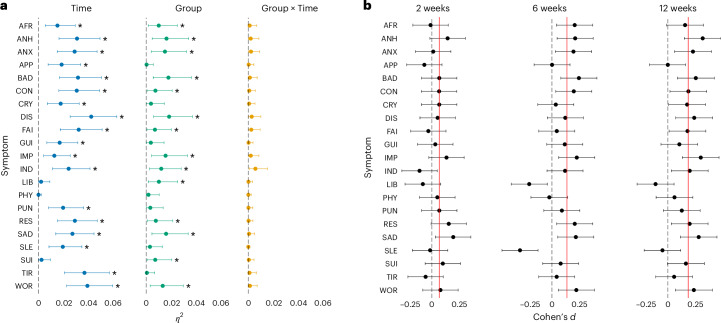
Effect sizes of the effect of sertraline on individual symptoms. **a**,**b**, Data are presented as effect sizes (*η*^2^) of time, treatment group and group by time interactions and associated confidence intervals (95% CI) in linear mixed models for each symptom. In these models, baseline scores for each symptom were included as fixed effects. Asterisks indicate a significant effect (based on FDR-corrected *P* values) (**a**). Data are presented as effect sizes (Cohen’s *d*) of sertraline on each symptom at 2, 6 and 12 weeks and associated confidence intervals (95% CI). Red lines indicate effect sizes derived from the main (sum-score) results of the PANDA trial (**b**).

A maximum sample of 571 individuals with complete cases for each symptom was included in this analysis (Supplementary Table 3). Mixed models indicated significant main effects of sertraline on all symptoms (accounting for baseline score), with small effect sizes (*η*^2^ = 0.007–0.019) (Figs. 1 and 2), except for problems with appetite, crying, feelings of guilt, physical health, feelings of self-punishment, sleep and tiredness. The largest beneficial effects of sertraline were on feelings of self-loathing (false discovery rate (FDR) *P* value, *P*_FDR_ < 0.001, *η*^2^ = 0.019, 95% confidence interval (CI) 0.006–0.038), feeling bad about oneself (*P*_FDR_ < 0.001, *η*^2^ = 0.018, 95% CI 0.006–0.037) and anhedonia (*P*_FDR_ < 0.001, *η*^2^ = 0.017, 95% CI 0.005–0.035). There were significant main effects of time on all symptoms except problems with libido, physical health, and suicidal thoughts (Supplementary Table 4). Following corrections for multiple comparisons, no treatment-by-time interactions achieved significance (Supplementary Table 4).

Standardized differences in means between sertraline and placebo groups (Cohen’s *d*) indicated comparable effect sizes to the main results of the PANDA trial, with larger effect sizes on somatic symptoms (for example, libido and sleep) at week 6 (Fig. 2b).

### Contemporaneous networks

**Fig. 3:**
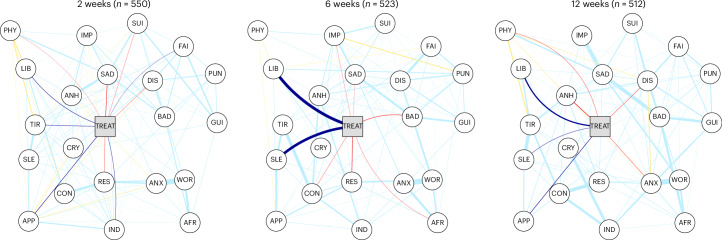
Contemporaneous networks of symptoms of depression and anxiety. In all networks, thicker edges indicate stronger associations. To highlight connections to the treatment node, positive associations (detrimental effects) with the treatment node (center) are in dark blue and negative associations (beneficial effects) in red. Positive associations between symptoms are in light blue and negative associations in yellow. Networks were plotted with an identical layout to better compare results. TREAT, treatment allocation.

We found beneficial effects of sertraline on symptoms across all assessments (*n*_2weeks_ = 550, *n*_6weeks_ = 523_,_ *n*_12weeks_ = 512) in contemporaneous networks (Fig. 3 and Supplementary Tables 5–7). Sertraline treatment caused lower feelings of sadness (*r*_2weeks_ = −0.092), restlessness (*r*_2weeks_ = −0.053), self-loathing (*r*_2weeks_ = −0.044), suicidal thoughts (*r*_2weeks_ = −0.039) and physical health problems (*r*_2weeks_ = −0.028) at the 2-week time point; lower levels of feeling bad about oneself (*r*_6weeks_ = −0.087), sadness (*r*_2weeks_ = −0.027), feeling afraid (*r*_6weeks_ = −0.041), restlessness (*r*_6weeks_ = −0.098) and concentration problems (*r*_6weeks_ = −0.0046) at the 6-week time point; and lower levels of anxiety (*r*_12weeks_ = −0.057), physical health problems (*r*_12weeks_ = −0.055), anhedonia (*r*_12weeks_ = −0.103) and self-loathing (*r*_12weeks_ = −0.061) at the 12-week time point. In addition, sertraline treatment caused higher self-reported improvement at 6 weeks (*r*_6weeks_ = −0.036). However, sertraline also had detrimental effects at all time points, such as on problems with sleep (*r*_6weeks_ = 0.219, *r*_12weeks_ = 0.065), appetite (*r*_2weeks_ = 0.089, *r*_12weeks_ = 0.099), libido (*r*_2weeks_ = 0.082, *r*_6weeks_ = 0.235, *r*_12weeks_ = 0.132), tiredness (*r*_2weeks_ = 0.077), fatigue (*r*_2weeks_ = 0.039) and indecisiveness (*r*_2weeks_ = 0.065).

#### Network structure comparison in contemporaneous networks

The network comparison test revealed no significant differences in network structure between placebo and sertraline networks (all *P* > 0.05).

### Temporally lagged networks

**Fig. 4:**
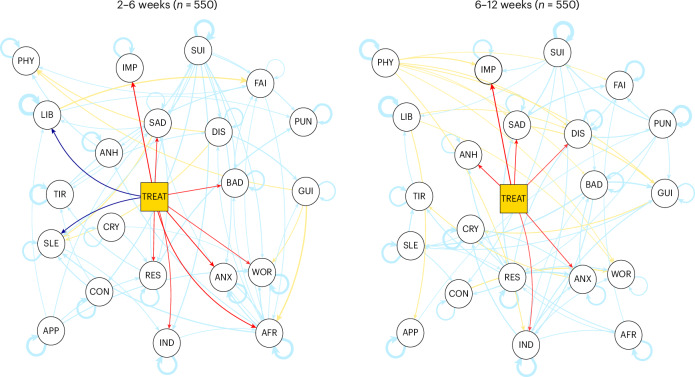
Temporally lagged networks of symptoms of depression and anxiety. Thicker edges indicate stronger associations. Directed arrows represent cross-lagged associations and looped arrows represent autoregressive associations. To highlight connections to the treatment node, positive associations (detrimental effects) with the treatment node (center) are in dark blue and negative associations (beneficial effects) in red. Positive associations between symptoms are in light blue and negative associations in yellow. Networks were plotted with an identical layout to better compare results.

The estimated cross-lagged panel model had adequate fit according to standard fit indices (comparative fit index (CFI) 0.965, root mean square error of approximation (RMSEA) 0.043). Sertraline caused lower symptoms of depression compared with placebo at all time points (*n* = 550) when controlling for temporal associations at previous time points (Fig. 4 and Supplementary Tables 8 and 9). For example, when accounting for symptoms at 2 weeks, sertraline caused, at 6 weeks, a reduction in feeling sad (*β*_6weeks_ = −0.096), bad about oneself (*β*_6weeks_ = −0.090), afraid (*β*_6weeks_ = −0.114), restlessness (*β*_6weeks_ = −0.091), anxiety (*β*_6weeks_ = −0.110), worry (*β*_6weeks_ = −0.083) and indecisiveness (*β*_6weeks_ = −0.086). Moreover, even when accounting for symptoms at 6 weeks, sertraline still caused, at 12 weeks, a reduction in feeling sad (*β*_12weeks_ = −0.106), anxiety (*β*_12weeks_ = −0.092), anhedonia (*β*_12weeks_ = −0.105), self-loathing (*β*_12weeks_ = −0.084) and indecisiveness (*β*_12weeks_ = −0.081). Notably, sertraline treatment consistently caused self-reported improvement over time (*β*_6weeks_ = −0.121, *β*_12weeks_ = −0.130) but also caused problems with libido (*β*_6weeks_ = 0.116) and sleep (*β*_6weeks_ = 0.113) during the middle of treatment.

#### Network structure comparison in temporally lagged networks

We found no significant structural network differences between sertraline and placebo groups. The cross-lagged model where edges were set to be equal across groups (model 1) had better support than the model where edges were free to vary across groups (model 2) (Bayesian information criterion (BIC)_Model1_ 57,444, BIC_Model2_ 61,960, Akaike information criterion (AIC)_Model1_ 49,298, AIC_Model2_ 50,013; Supplementary Table 10).

## Discussion

We examined the effects of sertraline on symptoms of anxiety and depression and their associations in a sample drawn from a large placebo-controlled randomized trial. First, we found beneficial effects of sertraline on most symptoms of depression and anxiety when using typical analytical approaches (linear mixed models). Second, by accounting for associations between symptoms in network analyses, we found early effects on core emotional and volitional symptoms of depression and all symptoms of anxiety at ~2 weeks of treatment. These early beneficial effects may be masked when outcomes are measured using a single summary score by concurrent detrimental effects on somatic symptoms, which were also clear at 2 weeks. Finally, we found no evidence of differences in patterns of associations between symptoms (network structures), either at each time point or across time, between treatment groups.

Adjusting for associations between symptoms showed that antidepressants may act more rapidly on some symptoms of depression than has previously been suggested using a single summary score of symptoms^5^. When accounting for associations at each time point (contemporaneous networks), we found a rapid, albeit small, effect of sertraline on sad mood compared with placebo, appearing at ~2 weeks. In addition, analyses that account for temporal associations (temporally lagged networks) suggested that sertraline caused a reduction in all included anxiety symptoms, which is consistent with the (sum-score) results of the PANDA trial. However, importantly, using temporally lagged networks, we found an additional clear reduction in core symptoms of depression, such as feeling sad and bad about oneself, at as early as 6 weeks. While these network findings are partially mirrored by typical analyses that do not account for associations between symptoms, the network results suggest sertraline may have an early effect on core symptoms of depression (such as sadness).

Importantly, our findings point to a pattern of contrasting effects of sertraline, with both beneficial and detrimental effects compared with placebo. Although the main results of the PANDA trial indicated no differences in adverse effects between groups, somatic symptoms of depression included in our analyses are also frequently reported side effects of SSRIs^5^. While we did not observe detrimental effects on sleep, tiredness and appetite when only examining symptoms individually (in linear mixed models), taking into consideration associations between symptoms provided additional sensitivity, revealing some detrimental effects of sertraline on libido, tiredness and appetite at as early at 2 weeks, and on sleep and libido at 6 weeks. However, we did not detect additional effects at 12 weeks of treatment beyond those at 6 weeks. By contrast, we found a continued beneficial effect of sertraline on depression and anxiety symptoms beyond 6 weeks of treatment, independent of its prior effects, and consistent with our predictions, on the basis of sum-score results of the PANDA trial. Therefore, the adverse impact on somatic symptoms may peak and stabilize within 6 weeks of continued sertraline administration, and it may be counteracted by improvements in other symptoms.

This pattern of opposing effects on symptoms would be overlooked in analyses using summary scores on depression scales (for example, the Beck Depression Inventory (BDI-II) and Patient Health Questionnaire (PHQ-9)) as primary outcomes. On the contrary, summary scores on anxiety scales (for example, the Generalized Anxiety Disorder Assessment (GAD-7)) may be more sensitive to certain improvements, as they may not include somatic symptoms associated with medication. Therefore, it is possible that the effects on depression reported in the primary analyses of the PANDA trial were partially attenuated by the inclusion of physiological indicators in main outcome measures (for example, sleep and appetite items in the PHQ-9). Finally, although we found direct effects of active treatment, we do not find evidence of different patterns of associations between symptoms across treatment groups (that is, differences in network structures). This suggests that antidepressant treatment may not alter the associations between symptoms. In other words, although sertraline may cause an improvement in core symptoms, it may not change their reciprocal associations. For example, sertraline may, on average, improve sad mood and worry, but it may not alter the extent to which these two symptoms are associated with each other. Therefore, sertraline does not seem to operate via interrupting maladaptive reinforcement cycles between symptoms.

Our findings align with the results of previous studies examining the effects of SSRIs on individual symptoms, which suggest that the likelihood of detecting an effect of an SSRI is greater when using depressed mood as the sole outcome (as opposed to sum-scores)^30^. Our findings also add to evidence that beneficial effects can be detected early in treatment^31^, along with detrimental effects on somatic symptoms^32^. In addition, our results are consistent with previous cross-sectional network studies indicating that SSRIs have effects on both affective and somatic symptoms^17,22,33^. We present a longitudinal, placebo-controlled analysis that captures associations between symptoms in a heterogeneous sample not typically included in randomized controlled trials, which provides an important demonstration of the above findings, in a population typical of that presenting to primary care for depression treatment. Importantly, our findings provide evidence of symptom-specific effects that generalize across trials and patient characteristics, suggesting that these effects reflect core features of the antidepressant response.

The interpretation of these findings has some limitations. First, psychological networks are dependent on the choice of network nodes^18,34,35^. Therefore, our findings are conditional on the selection of symptoms from commonly used depression and anxiety scales. However, the PHQ-9, BDI-II and GAD-7 include all the common symptoms of both depression and anxiety. Second, our findings should be further confirmed and replicated in independent samples. Third, some symptoms of depression and anxiety may be measured more reliably than others and are therefore more likely to be detected in network edges.

In conclusion, we show that sertraline has direct effects on individual anxiety and depression symptoms, as early as ~2 weeks into treatment, although it does not change associations between symptoms. Although the PANDA study found no evidence for an effect on depression at 6 weeks after starting sertraline, we observed effects of sertraline on depression symptoms at as early as 2 weeks. These beneficial effects may have been masked by detrimental effects on somatic symptoms such as libido and sleep. Using a network approach can reveal insights into the effectiveness, timing, and direct pathways of antidepressant action by taking into consideration individual symptoms and their associations.

## Methods

### Sample and measures

The sample included patients from the PANDA trial^29^ (Supplementary Table 1). In this trial, 653 adult patients (384 female, mean age 39.7 ± 14.96 years) with depressive symptoms were recruited in a primary care setting (ISRCTN ref. no. ISRCTN84544741). Participants received either sertraline—50 mg, once daily for 1 week, then 100 mg daily for up to 11 weeks—(*n* = 324, 203 female, mean age 39.7 ± 14.6 years) or placebo (*n* = 329, 181 female, mean age 39.7 ± 15.4 years), in a double-blind, randomized design. Details on recruitment, treatment allocation and randomization are described in detail by Lewis et al.^29^ and Salaminios et al.^36^. Ethics approval was obtained from the National Research Ethics Service Committee, East of England—Cambridge South (ref. no. 13/EE/0418). All participants provided written informed consent.

In the current analysis, we used the PHQ-9 (ref. ^37^), BDI-II (ref. ^38^) and GAD-7 (ref. ^39^) as measures of anxiety and depression symptoms; the physical health component of the Short Form Health Survey^40^; and a single item reflecting subjective improvement (‘Compared to 2 weeks ago, how have your moods and feelings changed?’, rated 1 for ‘I feel a lot better’ to 5 for ‘I feel a lot worse’). Depression severity was assessed with total scores on the Clinical Interview Schedule—Revised^41^, divided into three categories (0–11, 12–19 and ≥20). Patients were assessed at baseline and followed up at 2 weeks, 6 weeks, and 12 weeks after baseline.

### Statistical analysis

**Extended Data Fig. 1:**
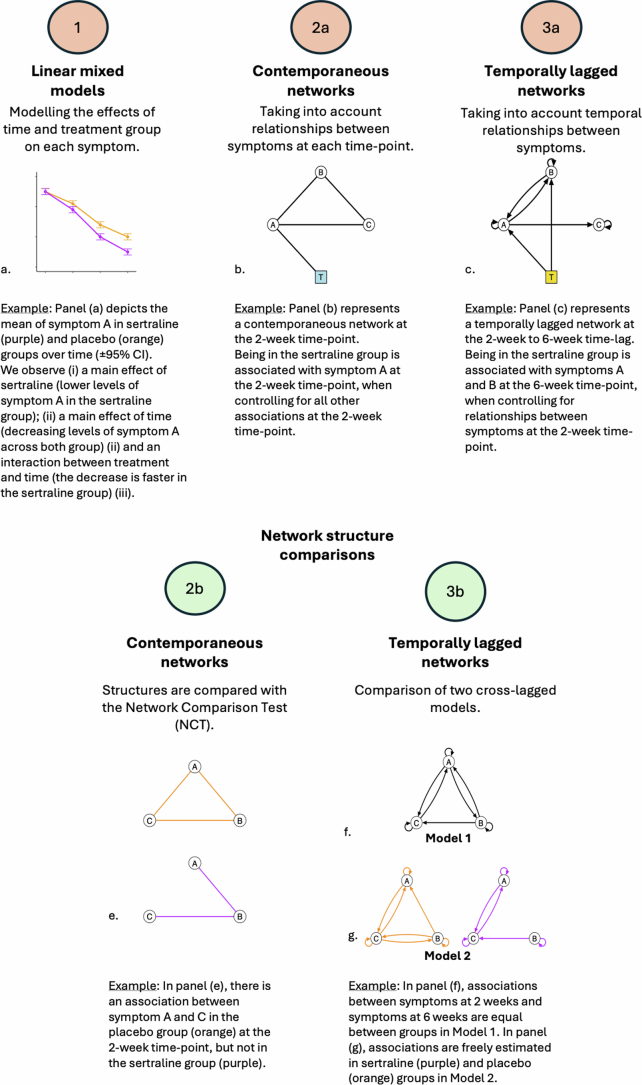
(a-g): Symptom-level analyses included in the study. Further discussed in Methods.

Analyses were carried out in R, version 4.2.0 (ref. ^42^) and are shown in Extended Data Fig. 1. Complete cases were used in each analysis step.

#### Node selection

To reduce the number of network nodes, both for interpretability and to avoid collinearity issues, we examined items of the selected scales for content overlap, using a combination of data-driven analysis and conceptual inspection of item similarity. First, using the ‘goldbricker’ function in the R package networktools^43^ (version 1.5.0), we identified correlated pairs of items that also showed a low proportion of statistically different correlations with other nodes (that is, variable pairs with correlations *r* ≥ 0.5 and <40% of significantly different correlations at *α* = 5% were flagged, using the ‘threshold’ argument in the goldbricker function). The identified pairs were then inspected for content overlap and, when appropriate, combined by taking mean values (rounded to the next integer; Supplementary Table 2). The selection procedure resulted in 21 symptoms.

#### Change in symptoms over time

We used standard linear mixed regression models to analyze the effects of time, treatment and their interaction on the 21 symptoms derived by node selection, using the R package lmerTest (version 3.1.3), restricted maximum likelihood estimation and Satterthwaite’s method for approximating degrees of freedom^44^. These models included time (2, 6 and 12 weeks) and individuals as random effects, allowing for random slopes. Site, the corresponding baseline symptom score, depression duration and treatment allocation were included as fixed effects, with an interaction between treatment and time. Effect sizes (*η*^2^, that is, the amount of variation in each item explained by predictors) and associated 95% CIs were obtained using the R package effectsize (version 0.7.0)^45^. *P* values were adjusted for multiple comparisons (21 tests) with FDR using the Benjamini–Hochberg method (*α* = 5%) and the R package stats (version 4.2.0)^42^. In addition, we calculated Cohen’s *d* for all symptoms at each time point and compared our estimates to the main PANDA trial results.

#### Network analyses

To compare our analyses with prior studies, we separately modeled each time point at which symptoms were measured (‘Contemporaneous networks’) (Extended Data Fig. 1). We then included associations between symptoms across time (‘Temporally lagged networks’). Within both network types, we modeled treatment allocation as a network node to estimate the direct effect of sertraline on individual symptoms while accounting for all other associations in a network. For example, we estimated the association between the treatment node and feelings of sadness, while accounting for all associations between symptoms. We then focused on a comparison of network structures between sertraline and placebo groups (‘Network structure comparisons’) in both contemporaneous and temporally lagged networks. This allowed us to establish whether individuals in either group had a greater number of nonzero associations between symptoms or showed stronger associations between symptoms. For example, we estimated whether there was a weaker association between feelings of sadness and low self-esteem in the sertraline group, relative to the placebo group, at the 2-week time point.

All item-level data used in networks were adjusted for covariates and baseline variables associated with missingness (identified in the main PANDA trial results) using linear regression models. In these models, each item was predicted by sex, age, surgery site, baseline item values, depression severity (Clinical Interview Schedule—Revised) and duration, ethnicity (‘White’ or ‘Ethnic minority’), financial difficulty (‘Comfortably/Alright’, ‘Just about coping’ or ‘Finding it difficult’), previous antidepressant use (‘Yes’ or ‘No’), marital status (‘Married/Living as married’, ‘Single’ or ‘Separated, divorced or widowed’) and notable life events (number of life events in the past 6 months). Standardized residuals obtained from linear regressions were then used in network analyses.

##### Contemporaneous networks

We estimated one network per time point using the mgm R package (version 1.2.13)^46^, modeling the selected symptoms and a node indicating treatment allocation (0 = placebo, 1 = sertraline)^47^. The least absolute shrinkage and selection operator was used to minimize the number of spurious edges, and cross-validation was used to select the least absolute shrinkage and selection operator tuning parameter (Supplementary Methods). In the resulting networks, edges represent partial correlations (*r*), and nodes represent symptoms at each time point. For network structure comparison in contemporaneous networks, we tested the null hypothesis that network edges were equal across sertraline and placebo groups for each contemporaneous network with a resampling-based permutation test (network comparison test with 1,000 iterations^48^).

##### Temporally lagged networks

We estimated a cross-lagged panel model including all symptoms (as observed variables) with the R package lavaan (version 0.6.12)^49^ using full information maximum likelihood estimation, including treatment allocation as a predictor^50^ (Supplementary Fig. 1). In this model, each symptom at one time point was regressed on all symptoms at the previous time point, allowing us to model the association of one symptom with another later symptom (cross-lagged paths) and with itself over time (autoregressive paths), while controlling for the associations with all other symptoms at the previous time point^51^. For example, we modeled the effect of concentration problems at the 2-week time point on sleep problems at the 6-week time point, while controlling for associations with all other symptoms at the 2-week time point.

The resulting standardized regression coefficients (*β*) were visualized as a network of directed edges. We report the model fit of the cross-lagged panel model according to standard fit indices (CFI and RMSEA, with CFI ≥0.95 and RMSEA ≤0.05 considered adequate model fit^52^).

For network structure comparison in temporally lagged networks, we compared groups by testing whether all edges between network nodes had comparable weights in the sertraline and placebo groups. We constructed a cross-lagged panel model without including treatment allocation as a variable (Supplementary Fig. 2). We then compared a model where all regression coefficients were set to be equal between groups (model 1) to a model where all coefficients were allowed to freely vary between groups (model 2) using common fit indices (CFI, RMSEA, AIC and BIC).

### Reporting summary

Further information on research design is available in the Nature Portfolio Reporting Summary linked to this article.

## Supplementary information

Supplementary Information Supplementary Tables 1–10 and Figs. 1 and 2.

Reporting Summary

## Data Availability

All de-identified individual participant data collected in the PANDA trial and related documents (study protocol, analysis plan and code) are available, with no end, from the publications of the original trial paper. To gain access, researchers will need to enter a data access agreement with University College London (London, UK), providing a proposal for the use of data and a request for access (glyn.lewis@ucl.ac.uk).

## Code availability

All code used for the analyses is available via GitHub at https://github.com/giuliapiazza18/PANDAnet-2/.
