# Supplementary material for: The effect of sertraline on networks of mood and anxiety symptoms: secondary analysis of the PANDA randomized controlled trial
Source: Nat Ment Health. 2025 Oct 30;3(11):1417–24. doi: 10.1038/s44220-025-00528-x (PMC12589115; doi:10.1038/s44220-025-00528-x)

nature mental health

Supplementary information

https://doi.org/10.1038/s44220-025-00528-x

The effect of sertraline on networks of mood and anxiety symptoms: secondary analysis of the PANDA randomized controlled trial

In the format provided by the authors and unedited

Table of Contents

Supplementary Methods *2*

Supplementary Tables *3*

Supplementary Table 1 3

Supplementary Table 2 4

Supplementary Table 3 6

Supplementary Table 4 7

Supplementary Table 5 10

Supplementary Table 6 13

Supplementary Table 7 16

Supplementary Table 8 19

Supplementary Table 9 19

Supplementary Table 10 41

Supplementary Figures *42*

Supplementary Figure 1 42

Supplementary Figure 2 43

Supplementary Methods

LASSO tuning parameter for contemporaneous network models

The LASSO tuning parameter (λ) was chosen based on k-fold cross validation with 10 folds. The sequence of tuning parameters was chosen based on the default procedure implemented in the *mgm* package (https://cran.r-project.org/web/packages/mgm/index.html), which relies on the *glmnet* package (https://cran.r-project.org/web/packages/glmnet/index.html). A sequence of λ values was determined from the data, starting from λ_{max} (the smallest value for which all coefficients are zero, ensuring sparsity) and λ_{min}, which is 0.0001 x λ_{max} when the number of observations is larger than the number of parameters to be estimated.

Bootstrapped CIs for contemporaneous network models

Confidence intervals (CIs) for contemporaneous networks were derived with non-parametric bootstrapping (2500 bootstraps).

Supplementary Tables

Supplementary Table 1

Demographic characteristics of the sample (size and percentage of the total sample) and means and standard deviations of age, total scores on PHQ-9 and GAD-7.

Characteristic	Sertraline	Placebo
	N = 324	N = 329
Sex		
Male	121 (37%)	148 (45%)
Female	203 (63%)	181 (55%)
Age	39.67 (15.38)	39.74 (14.56)
Ethnic Group		
White	294 (91%)	285 (87%)
Ethnic Minority	29 (9.0%)	44 (13%)
Site		
Bristol	131 (40%)	134 (41%)
Liverpool	58 (18%)	58 (18%)
York	66 (20%)	64 (19%)
London	69 (21%)	73 (22%)
Antidepressants used in the past	191 (59%)	200 (61%)
Depressed in the past	259 (80%)	263 (80%)
Highest qualifications		
Higher degree (e.g. M.A., PGCE) or equivalent	39 (12%)	42 (13%)
Degree (e.g. B. Sc., B.A.) or equivalent	67 (21%)	82 (25%)
Diploma (e.g. HND, NVQ, level 3) or equivalent	62 (19%)	61 (19%)
A-level or equivalent	48 (15%)	49 (15%)
GCSE, O-level, CSE or equivalent	76 (24%)	69 (21%)
Other qualifications	16 (5.0%)	8 (2.4%)
No qualifications	15 (4.6%)	18 (5.5%)
Marital Status		
Married/Living as married	116 (36%)	139 (42%)
Single	152 (47%)	144 (44%)
Separated	14 (4.3%)	14 (4.3%)
Divorced	30 (9.3%)	25 (7.6%)
Widowed	11 (3.4%)	7 (2.1%)
PHQ-9 total score	11.80 (5.89)	12.20 (5.71)
GAD-7 total score	9.44 (5.39)	9.42 (5.17)

Supplementary Table 2

Network nodes used in network estimation with brief description. Nodes were derived by combining questionnaire items from PHQ-9, GAD-7 and BDI-II.

Network node	Description	Questionnaire item(s)
BAD	Feeling bad about oneself	**PHQ-6** (“Feeling bad about yourself — or that you are a failure or have let yourself or your family down”)
ANX	Feeling nervous or anxious	**GAD-1** (“Feeling nervous, anxious, or on edge”)
AFR	Feeling afraid	**GAD-7** (“Feeling afraid, as if something awful might happen”)
FAI	Past failure	**BDI-3** (Past failure, from “I do not feel like a failure” to “I feel like I am a total failure as a person”)
GUI	Guilty feelings	**BDI -5** (Guilty feelings, from “I don’t feel particularly guilty” to “I feel guilty all of the time”)
PUN	Punishment feelings	**BDI -6** (Punishment feelings, from “I don’t feel I am being punished” to “I feel I am being punished”)
CRY	Crying	**BDI -10** (Crying, from “I don’t cry any more than I used to” to “I feel like crying, but I can’t”)
IND	Indecisiveness	**BDI -13** (Indecisiveness, from “I make decisions about as well as ever” to “I have trouble making any decisions”)
LIB	Loss of interest in sex	**BDI -21** (Loss of interest in sex, from “I have not noticed any recent change in my interest in sex” to “I have lost interest in sex completely”)
PHY	General physical health	**SF** physical scale
IMP	Self-reported improvement	-
TIR	Feeling tired	**BDI -20** (Tiredness of fatigue, from “I am no more tired or fatigued than usual” to “I am too tired or fatigued to do most of the things I used to do”), **BDI -15** (Loss of energy, from “I have as much energy as ever” to “I don’t have enough energy to do anything”), **PHQ-4** (“Feeling tired or having little energy”)
WOR	Feeling worried	**GAD-2** (“Not being able to stop or control worrying”), **GAD-3** (“Worrying too much about different things”)
ANH	Loss of interest and pleasure in everyday life	**BDI-12** (Loss of interest, from “I have not lost interest in other people or activities” to “It’s hard to get interested in anything”), **BDI-4** (“I get as much pleasure as I ever did from the things I enjoy” to “I can’t get any pleasure from the things I used to enjoy”), **PHQ-1** (“Little interest or pleasure in doing things”)
DIS	Disliking oneself	**BDI-14** (Worthlessness, from “I do not feel I am worthless” to “I feel utterly worthless”), **BDI-7** (Self-dislike, from “I feel the same about myself as ever” to “I dislike myself"), **BDI-8** (Self-criticalness, from “I don’t criticise myself or blame myself more than usual” to “I blame myself for everything bad that happens”), **BDI-2** (Pessimism, from “I am not discouraged about my future” to “I feel my future is hopeless and will only get worse”)
RES	Being restless or slow	**GAD-5** (“Being so restless that it is hard to sit still”), **GAD-6** (“Becoming easily annoyed or irritable”), **BDI-11** (Agitation, from “I am no more restless or wound up than usual” to “I am so restless or agitated that I have to keep moving or doing something”), **GAD-4** (“Trouble relaxing”), **PHQ-8** (“Moving or speaking so slowly that other people could have noticed, or the opposite, being so fidgety or restless that you have been moving around a lot more than usual”), **BDI-17** (Irritability, from “I am not more irritable than usual” to “I am irritable all the time”)

SUI	Suicidal thoughts	**BDI-9** (Suicidal thoughts or wishes, from "I don't have any thoughts of killing myself" to "I would kill myself if I had the chance"), **PHQ-9** ("Thoughts that you would be better off dead or hurting yourself in some way")
SAD	Feeling sad or depressed	**BDI-1** (Sadness, from "I do not feel sad" to "I am so sad or unhappy that I can't stand it"), **PHQ-2** ("Feeling down, depressed, or hopeless")
APP	Lack of appetite or eating too much	**BDI-18** (Changes in appetite, from "U have not experienced any change in my appetite" to "I have no appetite at all" to "I crave food all the time"), **PHQ-5** ("Poor appetite or overeating")
CON	Concentration problems	**BDI-19** (Concentration difficulty, from "I can concentrate as well as ever" to "I find I can't concentrate on anything"), **PHQ-7** ("Trouble concentrating on things, such as reading the newspaper or watching television")
SLE	Sleep problems	**BDI-16** (Changes in sleeping patterns, from "I have not experienced any change in my sleeping" to "I wake up 1-2 hours early and can't get back to sleep" or "I sleep most of the day"), **PHQ-3** ("Trouble falling asleep or staying asleep, or sleeping too much")

Supplementary Table 3

Sample size at each time point, for each group (placebo and sertraline) for each individual symptom included in networks.

**time**	**2wk**	**2wk**	**6wk**	**6wk**	**12wk**	**12wk**
**group**	placebo	sertraline	placebo	sertraline	placebo	sertraline
**BAD**	292	279	285	267	265	264
**ANX**	292	277	285	266	264	264
**AFR**	292	277	285	266	264	264
**FAI**	292	278	285	266	264	264
**GUI**	292	277	285	266	264	264
**PUN**	292	278	285	266	264	264
**CRY**	292	278	285	266	264	264
**IND**	292	278	285	266	264	264
**LIB**	289	274	285	266	260	260
**PHY**	291	275	277	254	264	263
**IMP**	292	279	285	267	265	264
**TIR**	292	279	285	267	265	264
**WOR**	292	277	285	266	264	264
**ANH**	292	279	285	267	265	264
**DIS**	292	278	285	266	264	264
**RES**	292	279	285	267	265	264
**SUI**	292	279	285	267	265	264
**SAD**	292	279	285	267	265	264
**APP**	292	279	285	267	265	264
**CON**	292	279	285	267	265	264
**SLE**	292	279	285	267	265	264

Supplementary Table 4

Results of linear mixed models

Df = Degrees of freedom; CI_low: lower confidence interval value, CI_high: higher confidence interval value

Symptom	Effect	Fvalue	Df	Eta	CI_low	CI_high	P value	Adjusted p value (FDR)
AFR	Group	11.14	1, 1071.9	0.01	0.002	0.026	0.001	0.002
AFR	Group x Time	1.65	1, 1517.5	0.001	0	0.007	0.2	0.466
AFR	Time	23.91	1, 1517	0.016	0.006	0.03	0	0
ANH	Group	18.19	1, 1075.6	0.017	0.005	0.035	0	0
ANH	Group x Time	3.04	1, 1571.5	0.002	0	0.009	0.081	0.285
ANH	Time	51.2	1, 1571.3	0.032	0.017	0.05	0	0
ANX	Group	16.68	1, 1070.2	0.015	0.004	0.033	0	0
ANX	Group x Time	3.43	1, 1566.3	0.002	0	0.009	0.064	0.285
ANX	Time	47.75	1, 1566.1	0.03	0.015	0.048	0	0
APP	Group	0.33	1, 1066.4	0	0	0.006	0.565	0.565
APP	Group x Time	0.47	1, 1551.6	0	0	0.004	0.493	0.69
APP	Time	29.95	1, 1551.2	0.019	0.008	0.034	0	0
BAD	Group	19.76	1, 1065.3	0.018	0.006	0.037	0	0
BAD	Group x Time	1.9	1, 1536.4	0.001	0	0.007	0.169	0.443
BAD	Time	51.36	1, 1536	0.032	0.017	0.052	0	0
CON	Group	8.14	1, 1069.4	0.008	0.001	0.021	0.004	0.008
CON	Group x Time	1.12	1, 1579.2	0.001	0	0.006	0.29	0.553
CON	Time	51.08	1, 1578.9	0.031	0.017	0.05	0	0
CRY	Group	4.17	1, 1058.8	0.004	0	0.015	0.041	0.058
CRY	Group x Time	0.98	1, 1578.8	0.001	0	0.005	0.321	0.56
CRY	Time	29.22	1, 1578.7	0.018	0.007	0.033	0	0
DIS	Group	20.54	1, 1073.2	0.019	0.006	0.038	0	0
DIS	Group x Time	4.46	1, 1607	0.003	0	0.01	0.035	0.285
DIS	Time	72.53	1, 1606.8	0.043	0.026	0.064	0	0
FAI	Group	7.72	1, 1072.7	0.007	0.001	0.021	0.006	0.008
FAI	Group x Time	3.77	1, 1567.6	0.002	0	0.01	0.052	0.285
FAI	Time	53.37	1, 1567.3	0.033	0.018	0.052	0	0

GUI	Group	4.06	1, 1065.7	0.004	0	0.015	0.044	0.058
GUI	Group x Time	0.27	1, 1569.4	0	0	0.004	0.601	0.749
GUI	Time	27.69	1, 1569.1	0.017	0.007	0.032	0	0
IMP	Group	17.32	1, 1073.7	0.016	0.004	0.034	0	0
IMP	Group x Time	3.27	1, 1628.2	0.002	0	0.009	0.071	0.285
IMP	Time	21.37	1, 1628.2	0.013	0.004	0.026	0	0
IND	Group	13.38	1, 1071.5	0.012	0.003	0.029	0	0.001
IND	Group x Time	8.75	1, 1507.3	0.006	0.001	0.016	0.003	0.066
IND	Time	38.15	1, 1507	0.025	0.012	0.042	0	0
LIB	Group	11.13	1, 1059.7	0.01	0.002	0.026	0.001	0.002
LIB	Group x Time	0.15	1, 1491.7	0	0	0.003	0.7	0.774
LIB	Time	3.16	1, 1491.4	0.002	0	0.009	0.076	0.079
PHY	Group	1.97	1, 1054.3	0.002	0	0.011	0.161	0.178
PHY	Group x Time	0.03	1, 1520.1	0	0	0.002	0.859	0.902
PHY	Time	0.03	1, 1520.1	0	0	0.002	0.864	0.864
PUN	Group	3.56	1, 1049.5	0.003	0	0.014	0.06	0.074
PUN	Group x Time	0.15	1, 1435.4	0	0	0.004	0.696	0.774
PUN	Time	29.51	1, 1435	0.02	0.008	0.037	0	0
RES	Group	8.52	1, 1070.9	0.008	0.001	0.022	0.004	0.007
RES	Group x Time	0.27	1, 1587.9	0	0	0.004	0.606	0.749
RES	Time	48.77	1, 1587.7	0.03	0.016	0.048	0	0
SAD	Group	17.81	1, 1075.2	0.016	0.005	0.034	0	0
SAD	Group x Time	0.89	1, 1614	0.001	0	0.005	0.347	0.56
SAD	Time	46.13	1, 1613.8	0.028	0.014	0.045	0	0
SLE	Group	3.3	1, 1070.1	0.003	0	0.013	0.07	0.081
SLE	Group x Time	0.01	1, 1587.1	0	0	0.001	0.934	0.934
SLE	Time	32.23	1, 1586.9	0.02	0.009	0.036	0	0
SUI	Group	7.97	1, 1070.2	0.007	0.001	0.021	0.005	0.008
SUI	Group x Time	0.59	1, 1596.8	0	0	0.005	0.442	0.662
SUI	Time	3.97	1, 1596.6	0.002	0	0.01	0.047	0.051
TIR	Group	0.61	1, 1066.1	0.001	0	0.007	0.436	0.457
TIR	Group x Time	1.47	1, 1534.1	0.001	0	0.007	0.225	0.472
TIR	Time	59.99	1, 1533.6	0.038	0.021	0.058	0	0

WOR	Group	14.66	1, 1069.7	0.014	0.003	0.03	0	0
WOR	Group x Time	2.02	1, 1555.8	0.001	0	0.007	0.156	0.443
WOR	Time	64.9	1, 1555.4	0.04	0.023	0.061	0	0

Supplementary Table 5

Contemporaneous network at week 2.

Node 1	Node 2	Edge estimate	Bootstrapped Lower CI	Bootstrapped Upper CI
AFR	APP	0.039	-0.043	0.121
AFR	CON	0.025	-0.048	0.099
AFR	GUI	0.073	-0.017	0.163
AFR	RES	0.067	-0.025	0.159
AFR	WOR	0.198	0.110	0.286
ANH	CON	0.049	-0.033	0.132
ANH	DIS	0.054	-0.032	0.139
ANH	SAD	0.204	0.123	0.285
ANH	SLE	0.111	0.024	0.199
ANH	SUI	0.031	-0.040	0.102
ANX	AFR	0.065	-0.033	0.164
ANX	ANH	-0.064	-0.163	0.035
ANX	APP	-0.096	-0.198	0.006
ANX	CON	0.051	-0.041	0.143
ANX	PUN	0.075	-0.023	0.172
ANX	RES	0.152	0.066	0.239
ANX	SAD	0.092	-0.004	0.187
ANX	WOR	0.374	0.293	0.455
APP	SLE	0.128	0.038	0.219
BAD	AFR	0.128	0.034	0.222
BAD	ANH	0.047	-0.033	0.127
BAD	DIS	0.138	0.045	0.231
BAD	FAI	0.123	0.040	0.205
BAD	IMP	0.109	0.026	0.192
BAD	PUN	0.097	-0.001	0.195
BAD	SAD	0.138	0.049	0.227
BAD	SUI	0.082	-0.009	0.173
BAD	TIR	0.028	-0.054	0.110
BAD	WOR	0.065	-0.031	0.160
CON	SLE	0.039	-0.043	0.120
CRY	ANH	0.064	-0.023	0.150
CRY	APP	-0.089	-0.187	0.009
CRY	CON	0.046	-0.037	0.128
CRY	DIS	0.104	0.012	0.196
CRY	IND	0.090	-0.012	0.192
CRY	LIB	0.081	-0.010	0.173
CRY	SUI	0.031	-0.050	0.111
CRY	TIR	0.060	-0.030	0.151
DIS	APP	0.055	-0.033	0.142
DIS	SAD	0.071	-0.015	0.157
DIS	SLE	0.054	-0.029	0.138
DIS	SUI	0.065	-0.028	0.157

FAI	APP	-0.057	-0.145	0.032
FAI	DIS	0.126	0.042	0.209
FAI	GUI	0.151	0.065	0.237
FAI	IND	0.030	-0.046	0.106
FAI	PUN	0.094	-0.003	0.191
FAI	SLE	0.061	-0.029	0.152
FAI	SUI	0.071	-0.012	0.155
FAI	TIR	0.095	0.008	0.181
GUI	CRY	0.027	-0.042	0.096
GUI	DIS	0.049	-0.036	0.135
GUI	IMP	0.026	-0.037	0.089
GUI	PUN	0.105	0.012	0.198
GUI	SAD	0.046	-0.031	0.122
GUI	SLE	0.063	-0.023	0.149
GUI	SUI	0.147	0.055	0.239
IMP	ANH	0.125	0.042	0.208
IMP	APP	0.064	-0.028	0.156
IMP	CON	0.056	-0.024	0.135
IMP	DIS	0.043	-0.035	0.121
IMP	SAD	0.131	0.043	0.218
IMP	SUI	0.050	-0.029	0.129
IMP	TIR	0.044	-0.040	0.127
IMP	WOR	0.062	-0.027	0.151
IND	ANH	0.097	0.009	0.186
IND	APP	0.138	0.051	0.225
IND	CON	0.105	0.009	0.201
IND	DIS	0.038	-0.044	0.119
IND	IMP	-0.038	-0.123	0.046
IND	LIB	0.054	-0.029	0.137
IND	RES	0.061	-0.026	0.149
IND	SAD	0.080	-0.011	0.171
IND	SLE	0.046	-0.038	0.130
IND	TIR	0.057	-0.028	0.142
LIB	ANH	0.086	-0.004	0.177
LIB	APP	0.060	-0.028	0.149
LIB	CON	0.133	0.046	0.219
LIB	IMP	0.031	-0.052	0.115
LIB	PHY	-0.124	-0.217	-0.030
LIB	SLE	0.043	-0.046	0.132
PHY	APP	-0.035	-0.120	0.051
PHY	CON	-0.055	-0.155	0.045
PHY	IMP	0.067	-0.031	0.166
PHY	SAD	0.137	0.040	0.233
PHY	TIR	-0.122	-0.209	-0.035
PUN	ANH	0.032	-0.048	0.113

PUN	DIS	0.123	0.029	0.217
PUN	RES	0.082	-0.011	0.175
RES	APP	0.097	0.004	0.190
RES	CON	0.212	0.123	0.300
RES	SAD	0.038	-0.040	0.116
RES	SLE	0.080	-0.001	0.162
SAD	CON	0.043	-0.037	0.123
SUI	APP	-0.040	-0.126	0.045
SUI	SAD	0.138	0.056	0.220
TIR	ANH	0.097	0.010	0.185
TIR	CON	0.083	-0.002	0.169
TIR	DIS	0.083	-0.007	0.173
TIR	RES	0.093	0.008	0.177
TIR	SAD	0.042	-0.047	0.131
TIR	SLE	0.188	0.103	0.273
TIR	SUI	-0.035	-0.127	0.056
TREAT	APP	0.089	-0.038	0.216
TREAT	DIS	-0.044	-0.152	0.065
TREAT	FAI	0.039	-0.084	0.162
TREAT	IND	0.065	-0.056	0.185
TREAT	LIB	0.082	-0.048	0.212
TREAT	PHY	-0.028	-0.143	0.086
TREAT	RES	-0.053	-0.168	0.063
TREAT	SAD	-0.092	-0.212	0.028
TREAT	SUI	-0.039	-0.144	0.065
TREAT	TIR	0.077	-0.051	0.205
WOR	ANH	0.080	-0.010	0.169
WOR	APP	0.059	-0.039	0.156
WOR	RES	0.144	0.060	0.228
WOR	SUI	0.078	-0.015	0.170

Supplementary Table 6

Contemporaneous network at week 6.

Node 1	Node 2	Edge estimate	Bootstrapped Lower CI	Bootstrapped Upper CI
AFR	ANH	0.031	-0.046	0.107
AFR	FAI	0.030	-0.055	0.114
AFR	PUN	0.058	-0.032	0.149
AFR	RES	0.096	0.006	0.187
AFR	SUI	0.030	-0.051	0.111
AFR	WOR	0.152	0.053	0.252
ANH	CON	0.148	0.063	0.233
ANH	DIS	0.118	0.031	0.205
ANH	SAD	0.112	0.017	0.207
ANX	AFR	0.201	0.102	0.300
ANX	RES	0.078	-0.016	0.172
ANX	SAD	0.114	0.012	0.216
ANX	WOR	0.368	0.277	0.458
APP	CON	0.045	-0.046	0.135
APP	SLE	0.196	0.109	0.283
BAD	ANH	0.080	-0.013	0.173
BAD	DIS	0.150	0.064	0.236
BAD	FAI	0.083	-0.018	0.185
BAD	GUI	0.174	0.077	0.270
BAD	IMP	0.043	-0.040	0.126
BAD	PUN	0.124	0.027	0.220
BAD	SAD	0.195	0.100	0.290
BAD	WOR	0.173	0.093	0.254
CON	SLE	0.077	-0.013	0.166
CRY	APP	0.071	-0.022	0.164
CRY	DIS	0.051	-0.034	0.136
CRY	IMP	0.042	-0.038	0.123
CRY	LIB	0.034	-0.045	0.113
CRY	SAD	0.081	-0.025	0.186
DIS	SAD	0.094	0.002	0.185
DIS	SUI	0.071	-0.022	0.163
FAI	DIS	0.209	0.124	0.295
FAI	GUI	0.061	-0.031	0.153
FAI	LIB	-0.050	-0.154	0.053
FAI	PUN	0.135	0.046	0.223
FAI	SAD	0.032	-0.049	0.113
FAI	SLE	0.092	-0.002	0.187
GUI	DIS	0.128	0.032	0.225
GUI	IND	0.084	-0.008	0.177
GUI	PUN	0.112	0.014	0.209

GUI	SAD	0.048	-0.033	0.130
GUI	SUI	0.038	-0.040	0.116
IMP	ANH	0.118	0.028	0.208
IMP	CON	0.079	-0.011	0.169
IMP	DIS	0.059	-0.027	0.144
IMP	RES	0.062	-0.023	0.147
IMP	SAD	0.164	0.077	0.251
IMP	SUI	0.139	0.042	0.236
IMP	WOR	0.053	-0.040	0.146
IND	ANH	0.143	0.048	0.239
IND	APP	0.079	-0.015	0.172
IND	CON	0.147	0.047	0.247
IND	DIS	0.067	-0.023	0.158
IND	RES	0.047	-0.035	0.129
IND	SUI	0.022	-0.053	0.097
IND	WOR	0.101	0.000	0.202
LIB	ANH	0.058	-0.035	0.151
LIB	APP	0.082	-0.014	0.178
LIB	PHY	-0.066	-0.159	0.027
LIB	RES	0.108	0.013	0.204
PHY	APP	-0.052	-0.141	0.037
PHY	IMP	0.103	0.001	0.205
PHY	SAD	0.064	-0.041	0.170
PUN	CON	0.081	-0.023	0.184
PUN	CRY	0.039	-0.047	0.125
PUN	DIS	0.141	0.057	0.225
PUN	IMP	-0.123	-0.225	-0.021
PUN	LIB	0.119	0.025	0.212
PUN	SUI	0.040	-0.055	0.134
RES	APP	0.141	0.052	0.229
RES	CON	0.117	0.035	0.200
RES	SAD	0.050	-0.034	0.134
RES	SLE	0.074	-0.015	0.164
RES	SUI	0.039	-0.044	0.123
SAD	CON	0.084	-0.008	0.176
SAD	SLE	0.039	-0.045	0.124
SUI	SAD	0.111	0.005	0.216
TIR	ANH	0.061	-0.027	0.150
TIR	APP	0.051	-0.041	0.142
TIR	CON	0.244	0.153	0.334
TIR	DIS	0.076	-0.013	0.166
TIR	RES	0.061	-0.025	0.146
TIR	SAD	0.033	-0.044	0.110
TIR	SLE	0.166	0.084	0.248
TIR	SUI	0.064	-0.023	0.151

TREAT	AFR	-0.041	-0.155	0.072
TREAT	BAD	-0.087	-0.219	0.045
TREAT	CON	-0.046	-0.163	0.071
TREAT	IMP	-0.036	-0.147	0.076
TREAT	LIB	0.235	0.103	0.367
TREAT	RES	-0.098	-0.230	0.033
TREAT	SAD	-0.027	-0.127	0.073
TREAT	SLE	0.219	0.085	0.353
WOR	APP	0.053	-0.039	0.145
WOR	RES	0.152	0.062	0.241
WOR	SAD	0.068	-0.033	0.169

Supplementary Table 7

Contemporaneous network at week 12.

Node 1	Node 2	Edge estimate	Bootstrapped Lower CI	Bootstrapped Upper CI
AFR	ANH	0.041	-0.051	0.133
AFR	CRY	0.068	-0.042	0.178
AFR	IND	0.055	-0.041	0.152
AFR	PUN	0.044	-0.057	0.145
AFR	RES	0.070	-0.033	0.172
AFR	TIR	0.047	-0.048	0.143
AFR	WOR	0.152	0.051	0.254
ANH	APP	0.103	0.004	0.202
ANH	CON	0.102	0.005	0.200
ANH	DIS	0.119	0.025	0.214
ANH	RES	0.113	0.021	0.206
ANH	SAD	0.088	-0.003	0.180
ANH	SLE	0.040	-0.046	0.127
ANH	SUI	0.049	-0.046	0.144
ANX	AFR	0.101	0.003	0.199
ANX	CRY	0.033	-0.052	0.117
ANX	DIS	-0.113	-0.243	0.016
ANX	FAI	0.091	-0.010	0.191
ANX	GUI	0.044	-0.047	0.135
ANX	IMP	0.064	-0.032	0.160
ANX	IND	0.070	-0.028	0.168
ANX	PHY	-0.058	-0.152	0.036
ANX	RES	0.114	0.014	0.214
ANX	SLE	0.068	-0.025	0.161
ANX	SUI	0.096	-0.003	0.195
ANX	WOR	0.305	0.223	0.386
APP	CON	0.113	0.013	0.214
APP	SLE	0.122	0.027	0.217
BAD	AFR	0.178	0.082	0.275
BAD	APP	0.069	-0.033	0.171
BAD	DIS	0.159	0.063	0.255
BAD	FAI	0.080	-0.026	0.185
BAD	GUI	0.179	0.087	0.272
BAD	PUN	0.095	-0.014	0.205
BAD	SAD	0.235	0.150	0.321
BAD	SUI	0.099	-0.005	0.202
BAD	WOR	0.105	0.012	0.198
CON	SLE	0.084	-0.018	0.186
CRY	APP	0.052	-0.038	0.142
CRY	DIS	0.078	-0.026	0.183

CRY	IND	0.191	0.089	0.293
CRY	LIB	0.044	-0.045	0.133
CRY	RES	0.098	0.001	0.196
CRY	TIR	0.061	-0.026	0.147
DIS	RES	0.095	-0.001	0.191
DIS	SAD	0.088	-0.016	0.193
DIS	SUI	0.142	0.049	0.235
FAI	ANH	0.056	-0.043	0.155
FAI	DIS	0.139	0.045	0.233
FAI	GUI	0.115	0.020	0.210
FAI	PUN	0.131	0.030	0.232
FAI	SUI	0.039	-0.044	0.122
FAI	WOR	0.050	-0.044	0.144
GUI	ANH	0.059	-0.032	0.150
GUI	DIS	0.126	0.024	0.228
GUI	IND	0.137	0.045	0.229
GUI	PUN	0.125	0.033	0.217
IMP	ANH	0.097	0.000	0.194
IMP	RES	0.038	-0.049	0.125
IMP	SAD	0.252	0.163	0.342
IMP	TIR	0.036	-0.051	0.124
IMP	WOR	0.046	-0.047	0.138
IND	CON	0.181	0.086	0.277
LIB	ANH	0.045	-0.049	0.140
LIB	RES	0.052	-0.051	0.155
LIB	SAD	0.066	-0.046	0.178
LIB	TIR	0.136	0.043	0.228
PHY	ANH	-0.037	-0.126	0.052
PHY	DIS	-0.084	-0.199	0.031
PHY	SAD	0.142	0.033	0.252
PHY	TIR	-0.129	-0.229	-0.028
PUN	ANH	0.082	-0.023	0.187
PUN	APP	0.032	-0.058	0.122
PUN	CRY	0.052	-0.052	0.156
PUN	DIS	0.072	-0.030	0.174
PUN	IND	0.072	-0.028	0.172
PUN	PHY	0.043	-0.050	0.135
PUN	SUI	0.060	-0.042	0.163
RES	CON	0.233	0.146	0.320
RES	SAD	0.091	-0.005	0.186
RES	SLE	0.059	-0.037	0.154
SAD	CON	0.076	-0.019	0.172
SUI	SAD	0.121	0.015	0.226
TIR	ANH	0.131	0.037	0.224
TIR	CON	0.150	0.055	0.246

TIR	RES	0.074	-0.019	0.167
TIR	SAD	0.084	-0.013	0.181
TIR	SLE	0.227	0.141	0.313
TREAT	ANH	-0.103	-0.235	0.030
TREAT	ANX	-0.057	-0.171	0.057
TREAT	APP	0.099	-0.038	0.236
TREAT	DIS	-0.061	-0.176	0.054
TREAT	LIB	0.132	-0.009	0.272
TREAT	PHY	-0.055	-0.177	0.068
TREAT	SLE	0.065	-0.063	0.193
WOR	DIS	0.074	-0.019	0.167
WOR	RES	0.071	-0.020	0.162
WOR	SAD	0.139	0.051	0.227

Supplementary Table 8
Temporally lagged network between 2 and 6 weeks.

Time 1 node	Time 2 node	Edge estimate	Lower CI	Upper CI
BAD	BAD	0.227	0.128	0.322
ANX	BAD	0.000	-0.061	0.136
AFR	BAD	0.097	0.009	0.184
FAI	BAD	0.000	-0.155	0.026
GUI	BAD	0.000	-0.110	0.067
PUN	BAD	0.000	-0.007	0.177
CRY	BAD	0.000	-0.086	0.081
IND	BAD	0.000	-0.085	0.098
LIB	BAD	0.000	-0.150	0.016
PHY	BAD	0.000	-0.045	0.116
IMP	BAD	0.000	-0.085	0.100
TIR	BAD	0.000	-0.035	0.152
WOR	BAD	0.000	-0.041	0.175
ANH	BAD	0.000	-0.116	0.081
DIS	BAD	0.000	-0.081	0.110
RES	BAD	0.000	-0.104	0.099
SUI	BAD	0.129	0.041	0.216
SAD	BAD	0.000	-0.071	0.138
APP	BAD	0.000	-0.098	0.072
CON	BAD	0.000	-0.004	0.181
SLE	BAD	0.000	-0.126	0.054
TREAT	BAD	-0.092	-0.339	-0.029
BAD	ANX	0.000	-0.043	0.156
ANX	ANX	0.268	0.169	0.372
AFR	ANX	0.149	0.059	0.239
FAI	ANX	0.000	-0.111	0.075
GUI	ANX	0.000	-0.159	0.024
PUN	ANX	0.000	-0.068	0.121
CRY	ANX	0.000	-0.163	0.010
IND	ANX	0.000	-0.060	0.127
LIB	ANX	0.000	-0.129	0.042
PHY	ANX	0.000	-0.120	0.046
IMP	ANX	0.000	-0.113	0.078
TIR	ANX	0.000	-0.088	0.105
WOR	ANX	0.000	-0.046	0.176
ANH	ANX	0.000	-0.145	0.058
DIS	ANX	0.000	-0.174	0.022
RES	ANX	0.000	-0.139	0.071
SUI	ANX	0.131	0.042	0.222

SAD	ANX	0.000	-0.128	0.088
APP	ANX	0.000	-0.013	0.162
CON	ANX	0.000	-0.042	0.147
SLE	ANX	0.000	-0.025	0.160
TREAT	ANX	-0.111	-0.382	-0.064
BAD	AFR	0.000	-0.109	0.084
ANX	AFR	0.134	0.037	0.233
AFR	AFR	0.361	0.273	0.447
FAI	AFR	0.107	0.018	0.198
GUI	AFR	-0.178	-0.266	-0.090
PUN	AFR	0.000	-0.055	0.129
CRY	AFR	0.000	-0.052	0.114
IND	AFR	0.096	0.006	0.188
LIB	AFR	0.000	-0.121	0.044
PHY	AFR	0.000	-0.080	0.080
IMP	AFR	0.000	-0.066	0.118
TIR	AFR	0.000	-0.123	0.064
WOR	AFR	0.000	-0.077	0.138
ANH	AFR	0.000	-0.093	0.103
DIS	AFR	0.000	-0.070	0.119
RES	AFR	0.000	-0.084	0.118
SUI	AFR	0.000	-0.003	0.172
SAD	AFR	0.000	-0.159	0.049
APP	AFR	0.000	-0.040	0.129
CON	AFR	0.000	-0.184	0.000
SLE	AFR	0.000	-0.078	0.101
TREAT	AFR	-0.115	-0.385	-0.077
BAD	FAI	0.104	0.006	0.200
ANX	FAI	0.000	-0.066	0.131
AFR	FAI	0.096	0.008	0.183
FAI	FAI	0.108	0.018	0.199
GUI	FAI	0.000	-0.064	0.114
PUN	FAI	0.000	-0.095	0.090
CRY	FAI	0.000	-0.043	0.124
IND	FAI	0.000	-0.109	0.073
LIB	FAI	-0.165	-0.248	-0.081
PHY	FAI	0.000	-0.140	0.022
IMP	FAI	0.000	-0.097	0.088
TIR	FAI	0.136	0.043	0.231
WOR	FAI	0.000	-0.083	0.132
ANH	FAI	0.000	-0.060	0.137
DIS	FAI	0.000	-0.020	0.170
RES	FAI	0.000	-0.049	0.154
SUI	FAI	0.095	0.008	0.183
SAD	FAI	0.000	-0.159	0.051

APP	FAI	0.000	-0.020	0.150
CON	FAI	0.000	-0.032	0.153
SLE	FAI	0.000	-0.079	0.101
TREAT	FAI	0.000	-0.164	0.145
BAD	GUI	0.000	-0.066	0.135
ANX	GUI	0.000	-0.008	0.196
AFR	GUI	0.092	0.001	0.182
FAI	GUI	0.000	-0.103	0.084
GUI	GUI	0.191	0.099	0.283
PUN	GUI	0.000	-0.096	0.096
CRY	GUI	0.000	-0.050	0.124
IND	GUI	0.000	-0.060	0.129
LIB	GUI	0.000	-0.118	0.055
PHY	GUI	0.000	-0.092	0.075
IMP	GUI	0.000	-0.160	0.032
TIR	GUI	0.000	-0.098	0.097
WOR	GUI	0.000	-0.041	0.182
ANH	GUI	0.000	-0.143	0.061
DIS	GUI	0.147	0.047	0.245
RES	GUI	0.000	-0.058	0.153
SUI	GUI	0.000	-0.091	0.091
SAD	GUI	0.000	-0.123	0.093
APP	GUI	0.000	-0.071	0.105
CON	GUI	0.000	-0.034	0.157
SLE	GUI	0.000	-0.123	0.064
TREAT	GUI	0.000	-0.239	0.082
BAD	PUN	0.000	-0.034	0.158
ANX	PUN	0.000	-0.137	0.058
AFR	PUN	0.000	-0.017	0.157
FAI	PUN	0.000	-0.076	0.104
GUI	PUN	0.000	-0.155	0.021
PUN	PUN	0.312	0.225	0.408
CRY	PUN	0.000	-0.148	0.019
IND	PUN	0.000	-0.165	0.016
LIB	PUN	0.102	0.021	0.186
PHY	PUN	0.000	-0.079	0.081
IMP	PUN	0.000	-0.114	0.070
TIR	PUN	0.000	-0.052	0.134
WOR	PUN	0.000	-0.069	0.145
ANH	PUN	0.000	-0.105	0.090
DIS	PUN	0.000	-0.033	0.157
RES	PUN	0.145	0.049	0.251
SUI	PUN	0.122	0.037	0.211
SAD	PUN	0.000	-0.074	0.134
APP	PUN	0.000	-0.066	0.103

CON	PUN	0.000	-0.099	0.085
SLE	PUN	0.000	-0.060	0.119
TREAT	PUN	0.000	-0.131	0.176
BAD	CRY	0.000	-0.158	0.052
ANX	CRY	0.000	-0.036	0.177
AFR	CRY	0.000	-0.031	0.158
FAI	CRY	0.000	-0.120	0.076
GUI	CRY	0.000	-0.050	0.141
PUN	CRY	0.000	-0.137	0.062
CRY	CRY	0.190	0.097	0.279
IND	CRY	0.000	-0.096	0.102
LIB	CRY	0.000	-0.063	0.117
PHY	CRY	0.000	-0.093	0.082
IMP	CRY	0.000	-0.116	0.084
TIR	CRY	0.000	-0.136	0.067
WOR	CRY	0.000	-0.090	0.144
ANH	CRY	0.000	-0.103	0.109
DIS	CRY	0.000	-0.125	0.081
RES	CRY	0.000	-0.055	0.165
SUI	CRY	0.000	-0.050	0.139
SAD	CRY	0.000	-0.073	0.153
APP	CRY	0.000	-0.014	0.170
CON	CRY	0.000	-0.105	0.095
SLE	CRY	0.000	-0.069	0.126
TREAT	CRY	0.000	-0.243	0.092
BAD	IND	0.000	-0.138	0.056
ANX	IND	0.000	-0.002	0.195
AFR	IND	0.000	-0.071	0.104
FAI	IND	0.000	-0.159	0.022
GUI	IND	0.000	-0.088	0.090
PUN	IND	0.000	-0.030	0.155
CRY	IND	0.000	-0.084	0.084
IND	IND	0.323	0.235	0.418
LIB	IND	0.000	-0.060	0.107
PHY	IND	0.000	-0.044	0.117
IMP	IND	0.000	-0.139	0.047
TIR	IND	0.000	-0.082	0.107
WOR	IND	0.000	-0.157	0.059
ANH	IND	0.000	-0.109	0.089
DIS	IND	0.000	-0.092	0.100
RES	IND	0.000	-0.068	0.136
SUI	IND	0.147	0.060	0.236
SAD	IND	0.000	-0.112	0.098
APP	IND	0.000	-0.007	0.164
CON	IND	0.000	-0.044	0.141

SLE	IND	0.108	0.017	0.198
TREAT	IND	-0.087	-0.330	-0.019
BAD	LIB	0.000	-0.057	0.130
ANX	LIB	0.000	-0.066	0.124
AFR	LIB	0.000	-0.081	0.088
FAI	LIB	0.000	-0.096	0.078
GUI	LIB	0.000	-0.119	0.052
PUN	LIB	0.000	-0.102	0.076
CRY	LIB	0.000	-0.050	0.112
IND	LIB	0.000	-0.134	0.041
LIB	LIB	0.475	0.395	0.555
PHY	LIB	0.000	-0.123	0.032
IMP	LIB	0.000	-0.132	0.046
TIR	LIB	0.000	-0.040	0.141
WOR	LIB	0.000	-0.178	0.029
ANH	LIB	0.000	-0.026	0.164
DIS	LIB	0.000	-0.168	0.015
RES	LIB	0.000	-0.086	0.110
SUI	LIB	0.000	-0.014	0.155
SAD	LIB	0.101	0.002	0.203
APP	LIB	0.094	0.013	0.176
CON	LIB	0.000	-0.131	0.047
SLE	LIB	0.000	-0.087	0.086
TREAT	LIB	0.115	0.082	0.380
BAD	PHY	0.000	-0.078	0.118
ANX	PHY	0.000	-0.067	0.131
AFR	PHY	0.000	-0.056	0.120
FAI	PHY	0.097	0.007	0.189
GUI	PHY	-0.116	-0.205	-0.027
PUN	PHY	0.000	-0.086	0.100
CRY	PHY	0.000	-0.068	0.100
IND	PHY	0.000	-0.065	0.121
LIB	PHY	0.000	-0.119	0.048
PHY	PHY	0.426	0.343	0.506
IMP	PHY	0.000	-0.129	0.058
TIR	PHY	0.000	-0.123	0.067
WOR	PHY	0.000	-0.168	0.049
ANH	PHY	0.123	0.024	0.222
DIS	PHY	-0.123	-0.219	-0.026
RES	PHY	0.000	-0.174	0.032
SUI	PHY	0.000	-0.087	0.090
SAD	PHY	0.000	-0.114	0.098
APP	PHY	0.000	-0.040	0.131
CON	PHY	0.000	-0.101	0.085
SLE	PHY	0.000	-0.150	0.032

TREAT	PHY	0.000	-0.172	0.140
BAD	IMP	0.000	-0.130	0.082
ANX	IMP	0.000	-0.096	0.119
AFR	IMP	0.000	-0.051	0.140
FAI	IMP	0.000	-0.108	0.089
GUI	IMP	0.000	-0.148	0.046
PUN	IMP	0.000	-0.185	0.017
CRY	IMP	0.000	-0.061	0.122
IND	IMP	0.000	-0.093	0.106
LIB	IMP	0.000	-0.164	0.018
PHY	IMP	0.000	-0.137	0.039
IMP	IMP	0.285	0.183	0.385
TIR	IMP	0.000	-0.050	0.155
WOR	IMP	0.000	-0.110	0.125
ANH	IMP	0.000	-0.193	0.022
DIS	IMP	0.000	-0.124	0.084
RES	IMP	0.000	-0.147	0.076
SUI	IMP	0.000	-0.091	0.100
SAD	IMP	0.000	-0.047	0.181
APP	IMP	0.000	-0.091	0.095
CON	IMP	0.000	-0.094	0.108
SLE	IMP	0.000	-0.087	0.110
TREAT	IMP	-0.122	-0.414	-0.076
BAD	TIR	0.000	-0.036	0.157
ANX	TIR	0.000	-0.081	0.115
AFR	TIR	0.000	-0.109	0.065
FAI	TIR	0.000	-0.094	0.086
GUI	TIR	0.000	-0.171	0.005
PUN	TIR	0.000	-0.061	0.123
CRY	TIR	0.000	-0.035	0.132
IND	TIR	0.000	-0.178	0.003
LIB	TIR	0.000	-0.113	0.052
PHY	TIR	0.000	-0.107	0.053
IMP	TIR	0.000	-0.183	0.001
TIR	TIR	0.334	0.241	0.428
WOR	TIR	0.000	-0.142	0.072
ANH	TIR	0.000	-0.069	0.127
DIS	TIR	0.000	-0.107	0.083
RES	TIR	0.124	0.024	0.226
SUI	TIR	0.087	0.000	0.174
SAD	TIR	0.000	-0.077	0.132
APP	TIR	0.000	-0.038	0.131
CON	TIR	0.098	0.006	0.189
SLE	TIR	0.000	-0.010	0.169
TREAT	TIR	0.000	-0.211	0.097

BAD	WOR	0.000	-0.134	0.060
ANX	WOR	0.111	0.014	0.210
AFR	WOR	0.152	0.065	0.240
FAI	WOR	0.000	-0.075	0.106
GUI	WOR	-0.108	-0.197	-0.020
PUN	WOR	0.000	-0.080	0.105
CRY	WOR	0.088	0.005	0.172
IND	WOR	0.000	-0.114	0.068
LIB	WOR	0.000	-0.102	0.064
PHY	WOR	0.000	-0.061	0.100
IMP	WOR	0.000	-0.071	0.114
TIR	WOR	0.000	-0.155	0.032
WOR	WOR	0.312	0.208	0.424
ANH	WOR	0.000	-0.108	0.089
DIS	WOR	0.000	-0.107	0.083
RES	WOR	0.000	-0.139	0.064
SUI	WOR	0.120	0.034	0.209
SAD	WOR	0.000	-0.144	0.065
APP	WOR	0.132	0.048	0.218
CON	WOR	0.000	-0.079	0.106
SLE	WOR	0.000	-0.075	0.105
TREAT	WOR	-0.084	-0.324	-0.015
BAD	ANH	0.000	-0.105	0.098
ANX	ANH	0.000	-0.033	0.173
AFR	ANH	0.125	0.034	0.218
FAI	ANH	0.000	-0.097	0.092
GUI	ANH	0.000	-0.132	0.053
PUN	ANH	0.000	-0.083	0.110
CRY	ANH	0.000	-0.033	0.142
IND	ANH	0.000	-0.078	0.113
LIB	ANH	0.000	-0.127	0.047
PHY	ANH	0.000	-0.077	0.091
IMP	ANH	0.000	-0.128	0.066
TIR	ANH	0.000	-0.078	0.119
WOR	ANH	0.000	-0.145	0.081
ANH	ANH	0.272	0.171	0.377
DIS	ANH	0.000	-0.105	0.094
RES	ANH	0.000	-0.044	0.169
SUI	ANH	0.099	0.009	0.192
SAD	ANH	0.000	-0.134	0.085
APP	ANH	0.000	-0.063	0.115
CON	ANH	0.000	-0.049	0.144
SLE	ANH	0.000	-0.086	0.103
TREAT	ANH	0.000	-0.302	0.022
BAD	DIS	0.000	-0.007	0.189

ANX	DIS	0.000	-0.079	0.120
AFR	DIS	0.103	0.013	0.191
FAI	DIS	0.000	-0.147	0.036
GUI	DIS	0.000	-0.129	0.050
PUN	DIS	0.000	-0.049	0.138
CRY	DIS	0.000	-0.050	0.120
IND	DIS	0.000	-0.076	0.108
LIB	DIS	0.000	-0.071	0.097
PHY	DIS	0.000	-0.073	0.090
IMP	DIS	0.000	-0.076	0.112
TIR	DIS	0.118	0.023	0.213
WOR	DIS	0.000	-0.178	0.040
ANH	DIS	0.000	-0.127	0.073
DIS	DIS	0.199	0.100	0.293
RES	DIS	0.000	-0.039	0.167
SUI	DIS	0.107	0.018	0.195
SAD	DIS	0.000	-0.042	0.170
APP	DIS	0.000	-0.106	0.067
CON	DIS	0.000	-0.057	0.130
SLE	DIS	0.000	-0.116	0.067
TREAT	DIS	0.000	-0.241	0.073
BAD	RES	0.000	-0.130	0.065
ANX	RES	0.000	-0.100	0.098
AFR	RES	0.000	-0.041	0.136
FAI	RES	0.000	-0.061	0.122
GUI	RES	0.000	-0.118	0.061
PUN	RES	0.000	-0.106	0.080
CRY	RES	0.116	0.030	0.199
IND	RES	0.000	-0.132	0.052
LIB	RES	0.000	-0.098	0.070
PHY	RES	0.000	-0.145	0.017
IMP	RES	0.000	-0.122	0.065
TIR	RES	0.000	-0.117	0.073
WOR	RES	0.000	-0.042	0.176
ANH	RES	0.000	-0.156	0.042
DIS	RES	0.000	-0.134	0.058
RES	RES	0.237	0.135	0.340
SUI	RES	0.000	-0.012	0.164
SAD	RES	0.118	0.012	0.224
APP	RES	0.117	0.030	0.202
CON	RES	0.000	-0.035	0.151
SLE	RES	0.000	-0.034	0.148
TREAT	RES	-0.093	-0.340	-0.027
BAD	SUI	0.000	-0.105	0.090
ANX	SUI	0.000	-0.131	0.067

AFR	SUI	0.000	-0.054	0.123
FAI	SUI	0.000	-0.058	0.124
GUI	SUI	0.000	-0.166	0.013
PUN	SUI	0.000	-0.072	0.114
CRY	SUI	0.000	-0.124	0.045
IND	SUI	0.000	-0.157	0.027
LIB	SUI	0.000	-0.104	0.064
PHY	SUI	0.000	-0.112	0.050
IMP	SUI	0.000	-0.134	0.053
TIR	SUI	0.000	-0.031	0.158
WOR	SUI	0.000	-0.124	0.094
ANH	SUI	0.000	-0.101	0.098
DIS	SUI	0.000	-0.063	0.130
RES	SUI	0.107	0.002	0.208
SUI	SUI	0.395	0.294	0.470
SAD	SUI	0.000	-0.126	0.084
APP	SUI	0.000	-0.009	0.162
CON	SUI	0.000	-0.080	0.106
SLE	SUI	0.000	-0.094	0.088
TREAT	SUI	0.000	-0.178	0.133
BAD	SAD	0.000	-0.049	0.150
ANX	SAD	0.000	-0.051	0.150
AFR	SAD	0.092	0.002	0.181
FAI	SAD	0.000	-0.048	0.137
GUI	SAD	0.000	-0.174	0.007
PUN	SAD	0.000	-0.063	0.126
CRY	SAD	0.000	-0.056	0.116
IND	SAD	0.000	-0.060	0.127
LIB	SAD	0.000	-0.160	0.010
PHY	SAD	0.000	-0.066	0.099
IMP	SAD	0.000	-0.016	0.173
TIR	SAD	0.000	-0.032	0.160
WOR	SAD	0.000	-0.087	0.133
ANH	SAD	0.000	-0.056	0.145
DIS	SAD	0.000	-0.172	0.024
RES	SAD	0.000	-0.135	0.073
SUI	SAD	0.127	0.037	0.216
SAD	SAD	0.139	0.034	0.248
APP	SAD	0.110	0.024	0.198
CON	SAD	0.000	-0.051	0.138
SLE	SAD	0.000	-0.128	0.056
TREAT	SAD	-0.096	-0.350	-0.034
BAD	APP	0.000	-0.117	0.081
ANX	APP	0.000	-0.118	0.084
AFR	APP	0.000	-0.176	0.003

FAI	APP	0.000	-0.159	0.026
GUI	APP	0.000	-0.038	0.143
PUN	APP	0.000	-0.010	0.178
CRY	APP	0.000	-0.041	0.131
IND	APP	0.000	-0.055	0.131
LIB	APP	0.000	-0.098	0.072
PHY	APP	0.000	-0.094	0.070
IMP	APP	0.000	-0.082	0.107
TIR	APP	0.000	-0.091	0.101
WOR	APP	0.000	-0.057	0.164
ANH	APP	0.000	-0.133	0.068
DIS	APP	0.000	-0.043	0.152
RES	APP	0.000	-0.044	0.164
SUI	APP	0.000	-0.055	0.124
SAD	APP	0.000	-0.196	0.018
APP	APP	0.344	0.260	0.434
CON	APP	0.000	-0.017	0.171
SLE	APP	0.000	-0.012	0.172
TREAT	APP	0.000	-0.124	0.192
BAD	CON	0.000	-0.079	0.120
ANX	CON	0.000	-0.066	0.136
AFR	CON	0.000	-0.023	0.157
FAI	CON	0.000	-0.150	0.035
GUI	CON	0.000	-0.113	0.069
PUN	CON	0.000	-0.082	0.107
CRY	CON	0.000	-0.040	0.132
IND	CON	0.000	-0.143	0.044
LIB	CON	0.000	-0.122	0.049
PHY	CON	0.000	-0.091	0.075
IMP	CON	0.000	-0.111	0.079
TIR	CON	0.000	-0.053	0.140
WOR	CON	0.000	-0.147	0.075
ANH	CON	0.000	-0.046	0.156
DIS	CON	0.000	-0.081	0.115
RES	CON	0.000	-0.046	0.163
SUI	CON	0.148	0.058	0.238
SAD	CON	0.000	-0.122	0.093
APP	CON	0.000	-0.001	0.174
CON	CON	0.281	0.186	0.375
SLE	CON	0.000	-0.089	0.096
TREAT	CON	0.000	-0.317	0.000
BAD	SLE	0.000	-0.118	0.077
ANX	SLE	0.000	-0.124	0.074
AFR	SLE	0.156	0.066	0.242
FAI	SLE	0.000	-0.079	0.103

GUI	SLE	0.000	-0.173	0.005
PUN	SLE	0.000	-0.079	0.106
CRY	SLE	0.000	-0.012	0.156
IND	SLE	0.000	-0.046	0.137
LIB	SLE	0.000	-0.098	0.069
PHY	SLE	0.000	-0.082	0.080
IMP	SLE	0.000	-0.070	0.116
TIR	SLE	0.000	-0.020	0.169
WOR	SLE	0.000	-0.020	0.197
ANH	SLE	0.000	-0.123	0.075
DIS	SLE	-0.119	-0.213	-0.022
RES	SLE	0.000	-0.100	0.104
SUI	SLE	0.098	0.010	0.186
SAD	SLE	-0.133	-0.239	-0.029
APP	SLE	0.000	-0.005	0.166
CON	SLE	0.140	0.046	0.231
SLE	SLE	0.249	0.155	0.336
TREAT	SLE	0.113	0.068	0.379

Supplementary Table 9
Temporally lagged network between 6 and 12 weeks.

Time 1 node	Time 2 node	Edge estimate	Lower CI	Upper CI
BAD	BAD	0.240	0.131	0.358
ANX	BAD	0.134	0.031	0.239
AFR	BAD	0.000	-0.026	0.159
FAI	BAD	0.000	-0.017	0.165
GUI	BAD	0.000	-0.068	0.114
PUN	BAD	0.138	0.049	0.228
CRY	BAD	0.000	-0.101	0.066
IND	BAD	0.096	0.006	0.188
LIB	BAD	0.000	-0.103	0.057
PHY	BAD	-0.082	-0.160	-0.007
IMP	BAD	0.000	-0.114	0.083
TIR	BAD	0.000	-0.038	0.153
WOR	BAD	0.000	-0.185	0.041
ANH	BAD	0.000	-0.152	0.046
DIS	BAD	0.000	-0.126	0.093
RES	BAD	0.000	-0.027	0.183
SUI	BAD	0.102	0.018	0.198
SAD	BAD	0.000	-0.071	0.161
APP	BAD	0.000	-0.116	0.060
CON	BAD	0.000	-0.130	0.075
SLE	BAD	0.000	-0.076	0.097
TREAT	BAD	0.000	-0.277	0.030
BAD	ANX	0.000	-0.017	0.219
ANX	ANX	0.240	0.133	0.350
AFR	ANX	0.000	-0.034	0.159
FAI	ANX	0.000	-0.055	0.135
GUI	ANX	0.000	-0.101	0.089
PUN	ANX	0.000	-0.104	0.083
CRY	ANX	0.119	0.035	0.209
IND	ANX	0.128	0.034	0.224
LIB	ANX	0.000	-0.057	0.109
PHY	ANX	0.000	-0.149	0.010
IMP	ANX	0.000	-0.067	0.138
TIR	ANX	0.000	-0.142	0.057
WOR	ANX	0.000	-0.183	0.052
ANH	ANX	0.000	-0.200	0.007
DIS	ANX	0.000	-0.155	0.072
RES	ANX	0.197	0.093	0.311
SUI	ANX	0.000	-0.004	0.184
SAD	ANX	0.000	-0.097	0.144

APP	ANX	0.000	-0.097	0.086
CON	ANX	0.000	-0.195	0.019
SLE	ANX	0.000	-0.059	0.121
TREAT	ANX	-0.087	-0.336	-0.016
BAD	AFR	0.000	-0.108	0.129
ANX	AFR	0.000	-0.099	0.119
AFR	AFR	0.310	0.215	0.407
FAI	AFR	0.000	-0.086	0.104
GUI	AFR	0.000	-0.014	0.176
PUN	AFR	0.000	-0.015	0.172
CRY	AFR	0.000	-0.075	0.099
IND	AFR	0.108	0.013	0.203
LIB	AFR	0.000	-0.117	0.050
PHY	AFR	0.000	-0.156	0.003
IMP	AFR	0.000	-0.113	0.092
TIR	AFR	0.000	-0.084	0.115
WOR	AFR	0.000	-0.061	0.174
ANH	AFR	0.000	-0.074	0.132
DIS	AFR	0.000	-0.137	0.091
RES	AFR	0.000	-0.023	0.196
SUI	AFR	0.000	-0.041	0.147
SAD	AFR	0.000	-0.142	0.099
APP	AFR	0.000	-0.134	0.050
CON	AFR	0.000	-0.153	0.061
SLE	AFR	0.000	-0.062	0.119
TREAT	AFR	0.000	-0.263	0.059
BAD	FAI	0.000	-0.072	0.154
ANX	FAI	0.000	-0.124	0.084
AFR	FAI	0.103	0.011	0.195
FAI	FAI	0.341	0.252	0.434
GUI	FAI	0.000	-0.063	0.119
PUN	FAI	0.143	0.052	0.232
CRY	FAI	0.000	-0.153	0.013
IND	FAI	0.000	-0.036	0.145
LIB	FAI	0.000	-0.085	0.074
PHY	FAI	-0.080	-0.156	-0.004
IMP	FAI	0.000	-0.160	0.037
TIR	FAI	0.000	-0.154	0.036
WOR	FAI	0.000	-0.135	0.090
ANH	FAI	0.000	-0.042	0.156
DIS	FAI	0.000	-0.116	0.101
RES	FAI	0.000	-0.052	0.157
SUI	FAI	0.000	-0.072	0.107
SAD	FAI	0.160	0.046	0.277
APP	FAI	0.000	-0.090	0.085

CON	FAI	0.000	-0.169	0.036
SLE	FAI	0.000	-0.043	0.130
TREAT	FAI	0.000	-0.279	0.027
BAD	GUI	0.183	0.066	0.306
ANX	GUI	0.000	-0.070	0.150
AFR	GUI	0.000	-0.139	0.056
FAI	GUI	0.000	-0.010	0.183
GUI	GUI	0.200	0.106	0.298
PUN	GUI	0.111	0.016	0.205
CRY	GUI	0.000	-0.053	0.123
IND	GUI	0.140	0.045	0.237
LIB	GUI	0.000	-0.161	0.007
PHY	GUI	-0.114	-0.196	-0.035
IMP	GUI	0.000	-0.077	0.130
TIR	GUI	0.000	-0.068	0.134
WOR	GUI	0.000	-0.118	0.120
ANH	GUI	0.000	-0.125	0.084
DIS	GUI	0.000	-0.040	0.190
RES	GUI	0.000	-0.022	0.199
SUI	GUI	0.000	-0.122	0.068
SAD	GUI	-0.136	-0.260	-0.015
APP	GUI	0.000	-0.136	0.050
CON	GUI	0.000	-0.119	0.098
SLE	GUI	0.000	-0.100	0.083
TREAT	GUI	0.000	-0.145	0.178
BAD	PUN	0.000	-0.106	0.125
ANX	PUN	0.000	-0.049	0.163
AFR	PUN	0.000	-0.025	0.163
FAI	PUN	0.000	-0.028	0.157
GUI	PUN	0.000	-0.032	0.154
PUN	PUN	0.434	0.347	0.530
CRY	PUN	0.000	-0.029	0.141
IND	PUN	0.000	-0.110	0.075
LIB	PUN	0.000	-0.152	0.010
PHY	PUN	0.000	-0.102	0.053
IMP	PUN	0.000	-0.184	0.016
TIR	PUN	0.000	-0.066	0.128
WOR	PUN	0.000	-0.129	0.101
ANH	PUN	0.000	-0.049	0.153
DIS	PUN	0.000	-0.076	0.146
RES	PUN	0.000	-0.149	0.064
SUI	PUN	0.000	-0.056	0.127
SAD	PUN	0.000	-0.174	0.062
APP	PUN	0.088	0.001	0.180
CON	PUN	0.000	-0.049	0.159

SLE	PUN	0.000	-0.120	0.056
TREAT	PUN	0.000	-0.207	0.105
BAD	CRY	0.000	-0.180	0.058
ANX	CRY	0.000	-0.157	0.061
AFR	CRY	0.131	0.034	0.227
FAI	CRY	0.000	-0.036	0.155
GUI	CRY	-0.124	-0.219	-0.028
PUN	CRY	0.000	-0.034	0.154
CRY	CRY	0.325	0.241	0.416
IND	CRY	0.146	0.050	0.240
LIB	CRY	0.000	-0.133	0.034
PHY	CRY	0.000	-0.144	0.016
IMP	CRY	0.000	-0.041	0.165
TIR	CRY	0.000	-0.056	0.144
WOR	CRY	0.000	-0.134	0.102
ANH	CRY	0.000	-0.052	0.156
DIS	CRY	0.000	-0.085	0.143
RES	CRY	0.000	-0.035	0.185
SUI	CRY	0.000	-0.132	0.057
SAD	CRY	0.000	-0.179	0.064
APP	CRY	0.000	-0.107	0.077
CON	CRY	0.000	-0.089	0.126
SLE	CRY	0.000	-0.007	0.174
TREAT	CRY	0.000	-0.301	0.021
BAD	IND	0.138	0.026	0.251
ANX	IND	0.000	-0.072	0.135
AFR	IND	0.000	-0.008	0.175
FAI	IND	0.000	-0.080	0.101
GUI	IND	0.000	-0.129	0.053
PUN	IND	0.000	-0.019	0.159
CRY	IND	0.000	-0.062	0.104
IND	IND	0.366	0.276	0.456
LIB	IND	0.000	-0.067	0.091
PHY	IND	-0.115	-0.191	-0.040
IMP	IND	0.000	-0.046	0.150
TIR	IND	0.000	-0.188	0.002
WOR	IND	0.000	-0.151	0.073
ANH	IND	0.000	-0.113	0.084
DIS	IND	0.000	-0.046	0.170
RES	IND	0.000	-0.059	0.149
SUI	IND	0.121	0.037	0.216
SAD	IND	0.000	-0.206	0.025
APP	IND	0.000	-0.054	0.121
CON	IND	0.000	-0.105	0.098
SLE	IND	0.000	-0.026	0.145

TREAT	IND	-0.077	-0.307	-0.002
BAD	LIB	0.000	-0.123	0.115
ANX	LIB	0.000	-0.190	0.028
AFR	LIB	0.000	-0.127	0.066
FAI	LIB	0.000	-0.038	0.153
GUI	LIB	0.000	-0.153	0.038
PUN	LIB	0.000	-0.120	0.069
CRY	LIB	0.000	-0.010	0.165
IND	LIB	0.000	-0.174	0.016
LIB	LIB	0.479	0.399	0.565
PHY	LIB	0.000	-0.153	0.007
IMP	LIB	0.000	-0.113	0.093
TIR	LIB	0.106	0.007	0.207
WOR	LIB	0.000	-0.044	0.191
ANH	LIB	0.000	-0.129	0.079
DIS	LIB	0.000	-0.159	0.069
RES	LIB	0.000	-0.051	0.167
SUI	LIB	0.093	0.003	0.192
SAD	LIB	0.000	-0.144	0.098
APP	LIB	0.000	-0.105	0.078
CON	LIB	0.000	-0.097	0.117
SLE	LIB	0.000	-0.045	0.136
TREAT	LIB	0.000	-0.199	0.121
BAD	PHY	0.000	-0.080	0.166
ANX	PHY	0.000	-0.114	0.112
AFR	PHY	0.000	-0.082	0.118
FAI	PHY	0.000	-0.060	0.138
GUI	PHY	0.000	-0.161	0.037
PUN	PHY	0.000	-0.060	0.136
CRY	PHY	0.000	-0.133	0.047
IND	PHY	0.000	-0.049	0.148
LIB	PHY	0.000	-0.133	0.040
PHY	PHY	0.434	0.356	0.521
IMP	PHY	0.000	-0.049	0.165
TIR	PHY	0.000	-0.079	0.128
WOR	PHY	0.000	-0.078	0.167
ANH	PHY	0.000	-0.130	0.086
DIS	PHY	0.000	-0.209	0.028
RES	PHY	0.000	-0.185	0.042
SUI	PHY	0.000	-0.060	0.135
SAD	PHY	0.000	-0.189	0.062
APP	PHY	0.000	-0.138	0.053
CON	PHY	0.000	-0.116	0.106
SLE	PHY	0.000	-0.162	0.026
TREAT	PHY	0.000	-0.295	0.039

BAD	IMP	0.000	-0.119	0.143
ANX	IMP	0.000	-0.059	0.182
AFR	IMP	0.000	-0.089	0.125
FAI	IMP	0.000	-0.036	0.175
GUI	IMP	0.000	-0.166	0.044
PUN	IMP	0.000	-0.078	0.130
CRY	IMP	0.000	-0.098	0.095
IND	IMP	0.000	-0.115	0.095
LIB	IMP	0.000	-0.128	0.057
PHY	IMP	-0.148	-0.237	-0.061
IMP	IMP	0.267	0.157	0.385
TIR	IMP	0.000	-0.046	0.175
WOR	IMP	0.000	-0.133	0.128
ANH	IMP	0.000	-0.208	0.022
DIS	IMP	0.000	-0.211	0.042
RES	IMP	0.000	-0.067	0.175
SUI	IMP	0.116	0.017	0.225
SAD	IMP	0.000	-0.168	0.101
APP	IMP	0.000	-0.073	0.131
CON	IMP	0.000	-0.202	0.036
SLE	IMP	0.000	-0.099	0.101
TREAT	IMP	-0.130	-0.441	-0.086
BAD	TIR	0.000	-0.085	0.153
ANX	TIR	-0.116	-0.225	-0.006
AFR	TIR	0.000	-0.024	0.171
FAI	TIR	0.000	-0.158	0.034
GUI	TIR	0.000	-0.048	0.144
PUN	TIR	0.000	-0.003	0.186
CRY	TIR	0.000	-0.017	0.158
IND	TIR	0.000	-0.092	0.099
LIB	TIR	0.000	-0.061	0.106
PHY	TIR	0.000	-0.132	0.029
IMP	TIR	0.000	-0.087	0.120
TIR	TIR	0.260	0.160	0.361
WOR	TIR	0.000	-0.101	0.136
ANH	TIR	0.000	-0.073	0.136
DIS	TIR	0.000	-0.150	0.080
RES	TIR	0.136	0.028	0.248
SUI	TIR	0.000	-0.055	0.134
SAD	TIR	0.000	-0.162	0.082
APP	TIR	0.000	-0.071	0.114
CON	TIR	0.000	-0.125	0.091
SLE	TIR	0.151	0.061	0.243
TREAT	TIR	0.000	-0.249	0.073
BAD	WOR	0.000	-0.009	0.220

ANX	WOR	0.000	-0.026	0.185
AFR	WOR	0.000	-0.039	0.147
FAI	WOR	0.000	-0.084	0.101
GUI	WOR	0.000	-0.107	0.077
PUN	WOR	0.000	-0.042	0.140
CRY	WOR	0.000	-0.011	0.157
IND	WOR	0.107	0.015	0.199
LIB	WOR	-0.092	-0.173	-0.012
PHY	WOR	-0.091	-0.169	-0.015
IMP	WOR	0.000	-0.015	0.184
TIR	WOR	0.000	-0.063	0.130
WOR	WOR	0.149	0.035	0.263
ANH	WOR	0.000	-0.136	0.064
DIS	WOR	0.000	-0.115	0.105
RES	WOR	0.148	0.046	0.257
SUI	WOR	0.000	-0.052	0.129
SAD	WOR	0.000	-0.031	0.203
APP	WOR	0.000	-0.081	0.097
CON	WOR	-0.151	-0.257	-0.050
SLE	WOR	0.000	-0.074	0.100
TREAT	WOR	0.000	-0.256	0.055
BAD	ANH	0.000	-0.144	0.093
ANX	ANH	0.000	-0.098	0.119
AFR	ANH	0.000	-0.005	0.188
FAI	ANH	0.000	-0.021	0.170
GUI	ANH	0.000	-0.060	0.131
PUN	ANH	0.000	-0.011	0.177
CRY	ANH	0.000	-0.061	0.113
IND	ANH	0.000	-0.108	0.082
LIB	ANH	0.000	-0.105	0.062
PHY	ANH	0.000	-0.134	0.025
IMP	ANH	0.000	-0.048	0.158
TIR	ANH	0.000	-0.002	0.197
WOR	ANH	0.000	-0.145	0.091
ANH	ANH	0.190	0.087	0.294
DIS	ANH	0.000	-0.061	0.167
RES	ANH	0.145	0.040	0.259
SUI	ANH	0.000	-0.028	0.160
SAD	ANH	0.000	-0.178	0.065
APP	ANH	0.000	-0.084	0.100
CON	ANH	0.000	-0.139	0.075
SLE	ANH	0.000	-0.053	0.128
TREAT	ANH	-0.097	-0.358	-0.037
BAD	DIS	0.000	-0.107	0.120
ANX	DIS	0.000	-0.183	0.026

AFR	DIS	0.000	-0.013	0.172
FAI	DIS	0.112	0.023	0.206
GUI	DIS	0.000	-0.079	0.104
PUN	DIS	0.138	0.048	0.229
CRY	DIS	0.000	-0.086	0.081
IND	DIS	0.000	-0.068	0.114
LIB	DIS	-0.103	-0.184	-0.024
PHY	DIS	-0.114	-0.192	-0.039
IMP	DIS	0.000	-0.051	0.147
TIR	DIS	0.000	-0.074	0.117
WOR	DIS	0.000	-0.141	0.086
ANH	DIS	0.000	-0.092	0.107
DIS	DIS	0.289	0.186	0.404
RES	DIS	0.131	0.030	0.240
SUI	DIS	0.000	-0.037	0.144
SAD	DIS	0.000	-0.128	0.105
APP	DIS	0.000	-0.075	0.101
CON	DIS	0.000	-0.077	0.129
SLE	DIS	0.000	-0.091	0.082
TREAT	DIS	-0.078	-0.313	-0.005
BAD	RES	0.000	-0.153	0.078
ANX	RES	0.000	-0.121	0.091
AFR	RES	0.101	0.008	0.196
FAI	RES	0.000	-0.057	0.128
GUI	RES	0.000	-0.090	0.095
PUN	RES	0.103	0.011	0.194
CRY	RES	0.093	0.011	0.180
IND	RES	0.000	-0.041	0.144
LIB	RES	0.000	-0.136	0.026
PHY	RES	0.000	-0.106	0.049
IMP	RES	0.000	-0.096	0.105
TIR	RES	0.000	-0.012	0.182
WOR	RES	0.000	-0.104	0.125
ANH	RES	0.000	-0.144	0.058
DIS	RES	0.000	-0.079	0.143
RES	RES	0.313	0.216	0.429
SUI	RES	0.000	-0.017	0.165
SAD	RES	0.000	-0.184	0.052
APP	RES	0.000	-0.092	0.087
CON	RES	0.000	-0.051	0.158
SLE	RES	0.087	0.001	0.177
TREAT	RES	0.000	-0.251	0.062
BAD	SUI	0.000	-0.133	0.095
ANX	SUI	0.000	-0.079	0.129
AFR	SUI	0.000	-0.050	0.135

FAI	SUI	0.000	-0.110	0.073
GUI	SUI	0.000	-0.115	0.067
PUN	SUI	0.156	0.066	0.246
CRY	SUI	0.000	-0.125	0.042
IND	SUI	0.000	-0.063	0.119
LIB	SUI	0.000	-0.069	0.090
PHY	SUI	0.000	-0.086	0.066
IMP	SUI	0.000	-0.086	0.112
TIR	SUI	0.000	-0.143	0.048
WOR	SUI	0.000	-0.089	0.137
ANH	SUI	0.000	-0.088	0.111
DIS	SUI	0.000	-0.075	0.143
RES	SUI	0.000	-0.079	0.131
SUI	SUI	0.513	0.449	0.628
SAD	SUI	0.000	-0.112	0.120
APP	SUI	0.000	-0.077	0.099
CON	SUI	0.000	-0.133	0.073
SLE	SUI	0.000	-0.108	0.066
TREAT	SUI	0.000	-0.263	0.042
BAD	SAD	0.000	-0.067	0.168
ANX	SAD	0.000	-0.083	0.132
AFR	SAD	0.000	-0.093	0.098
FAI	SAD	0.000	-0.028	0.161
GUI	SAD	0.000	-0.111	0.078
PUN	SAD	0.000	-0.035	0.151
CRY	SAD	0.000	-0.095	0.078
IND	SAD	0.000	-0.092	0.096
LIB	SAD	0.000	-0.103	0.062
PHY	SAD	-0.085	-0.164	-0.006
IMP	SAD	0.000	-0.051	0.153
TIR	SAD	0.000	-0.041	0.157
WOR	SAD	0.000	-0.104	0.130
ANH	SAD	0.000	-0.155	0.051
DIS	SAD	0.000	-0.133	0.093
RES	SAD	0.158	0.052	0.269
SUI	SAD	0.149	0.061	0.248
SAD	SAD	0.213	0.095	0.335
APP	SAD	0.000	-0.096	0.087
CON	SAD	0.000	-0.147	0.065
SLE	SAD	0.000	-0.067	0.112
TREAT	SAD	-0.098	-0.356	-0.039
BAD	APP	0.000	-0.109	0.139
ANX	APP	0.000	-0.078	0.149
AFR	APP	0.000	-0.087	0.115
FAI	APP	0.000	-0.111	0.089

GUI	APP	0.000	-0.134	0.065
PUN	APP	0.000	-0.056	0.140
CRY	APP	0.000	-0.141	0.042
IND	APP	0.000	-0.086	0.113
LIB	APP	-0.127	-0.215	-0.040
PHY	APP	0.000	-0.153	0.014
IMP	APP	0.000	-0.158	0.057
TIR	APP	0.000	-0.096	0.112
WOR	APP	0.000	-0.083	0.163
ANH	APP	0.000	-0.047	0.170
DIS	APP	0.000	-0.128	0.111
RES	APP	0.000	-0.028	0.201
SUI	APP	0.000	-0.039	0.158
SAD	APP	0.000	-0.114	0.139
APP	APP	0.358	0.262	0.455
CON	APP	0.000	-0.157	0.067
SLE	APP	0.000	-0.025	0.164
TREAT	APP	0.000	-0.105	0.231
BAD	CON	0.000	-0.034	0.192
ANX	CON	0.000	-0.029	0.179
AFR	CON	0.000	-0.039	0.145
FAI	CON	0.000	-0.133	0.049
GUI	CON	0.000	-0.045	0.136
PUN	CON	0.000	-0.089	0.091
CRY	CON	0.000	-0.059	0.108
IND	CON	0.000	-0.010	0.172
LIB	CON	0.000	-0.124	0.035
PHY	CON	0.000	-0.138	0.015
IMP	CON	0.000	-0.148	0.049
TIR	CON	0.000	-0.066	0.124
WOR	CON	0.000	-0.214	0.011
ANH	CON	0.000	-0.106	0.092
DIS	CON	0.000	-0.074	0.144
RES	CON	0.159	0.057	0.266
SUI	CON	0.116	0.031	0.211
SAD	CON	0.000	-0.099	0.133
APP	CON	0.000	-0.040	0.136
CON	CON	0.201	0.100	0.305
SLE	CON	0.119	0.034	0.207
TREAT	CON	0.000	-0.216	0.091
BAD	SLE	0.172	0.053	0.294
ANX	SLE	0.000	-0.068	0.153
AFR	SLE	0.000	-0.089	0.107
FAI	SLE	0.000	-0.143	0.051
GUI	SLE	0.119	0.023	0.216

PUN	SLE	0.000	-0.034	0.157
CRY	SLE	0.090	0.003	0.180
IND	SLE	0.000	-0.047	0.147
LIB	SLE	0.000	-0.155	0.014
PHY	SLE	0.000	-0.145	0.017
IMP	SLE	0.000	-0.064	0.145
TIR	SLE	0.000	-0.009	0.194
WOR	SLE	0.000	-0.144	0.096
ANH	SLE	0.000	-0.115	0.096
DIS	SLE	0.000	-0.227	0.005
RES	SLE	0.000	-0.038	0.185
SUI	SLE	0.000	-0.082	0.109
SAD	SLE	0.000	-0.223	0.023
APP	SLE	0.000	-0.048	0.139
CON	SLE	0.000	-0.168	0.050
SLE	SLE	0.313	0.225	0.409
TREAT	SLE	0.000	-0.147	0.180

Supplementary Table 10

Comparison of network edges between sertraline and placebo groups in temporally lagged networks.

Model fit indices and Chi-square comparison for Model 1, where all edges were set to be equal between sertraline and placebo groups, and Model 2, where edges were freely estimated; Df: Degrees of Freedom; AIC: Akaike Information Criterion; BIC: Bayesian Information Criterion; CFI: Comparative Fit Index; RMSEA: Root Mean Square Error of Approximation.

	CFI	RMSEA	AIC	BIC	X^2	X^2 difference	Df	Df difference	p-value
**Model 2** *Different edges across groups*	0.950	0.052	50013	61960	1547		882		
**Model 1** *Equal edges across groups*	0.937	0.041	49298	57444	2596	1048	1764	882	0.00009

Supplementary Figures

Supplementary Figure 1

Schematic representation of the cross-lagged panel model used to derive network edges in Figure 3a-b.

In this model, treatment allocation is a time-invariant predictor ("TREAT"), predicting individual symptoms (observed variables) at 2-, 6- and 12-weeks follow-ups. In this example, A and B are individual symptoms, paths marked 't' are regression coefficients between treatment allocation and symptoms, 'a' paths indicate autoregressive relationships and 'c' paths indicate cross-lagged relationships. The full model includes 21 symptoms.

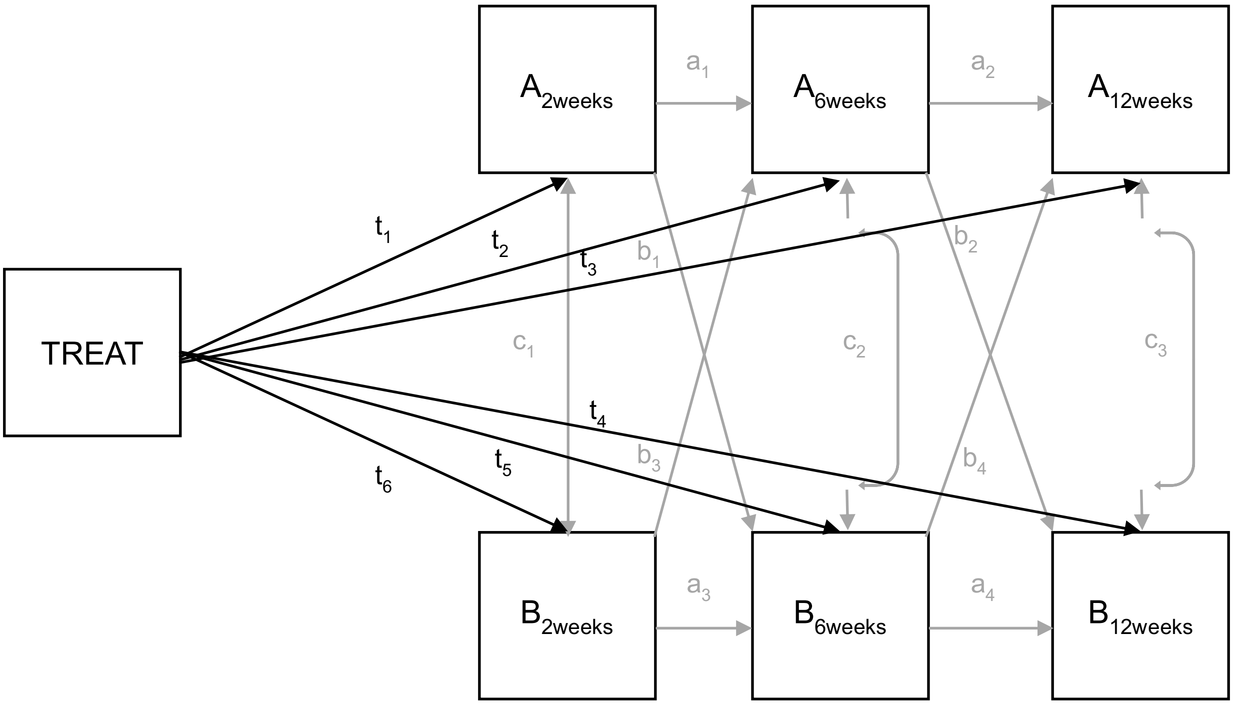

Supplementary Figure 2

Schematic representation of the cross-lagged panel model used to derive networks in Model 1-2.

In this example, A and B are individual symptoms (observed variables), paths marked ‘a’ indicate autoregressive relationships and ‘c’ paths indicate cross-lagged relationships. The full model includes 21 symptoms. Treatment and placebo groups were compared with multi-group modelling by fixing all paths to be equal between groups at the same time (Model 1). Comparisons of edges were then carried out by comparing a model with equal edges between groups (Model 1) to a model where edges were free to vary between groups (Models 2).

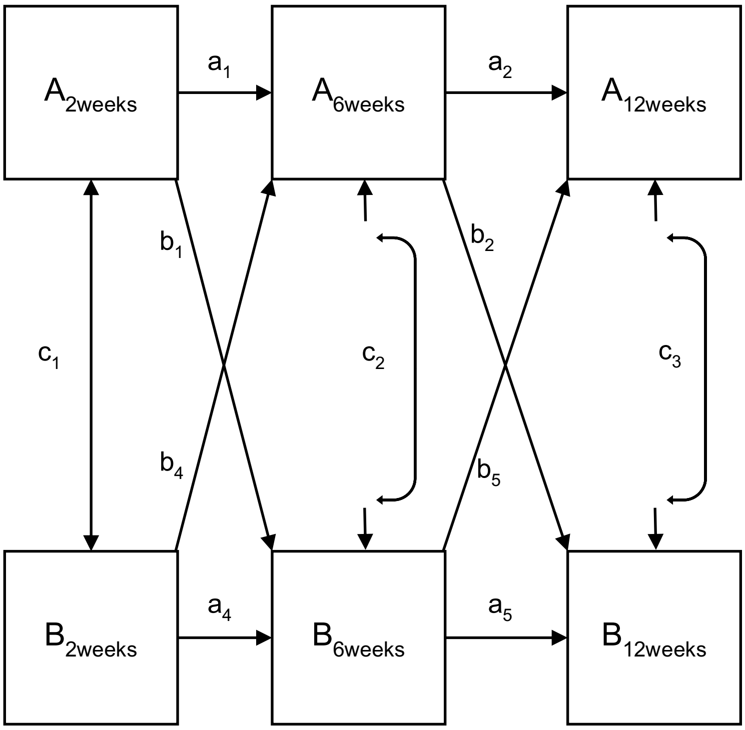

Supplement: Supplementary file 1 — Supplementary Tables 1–10 and Figs. 1 and 2. [file 44220_2025_528_MOESM1_ESM.pdf]
