# Supplementary material for: The effect of sertraline on networks of mood and anxiety symptoms: secondary analysis of the PANDA randomized controlled trial
Source: Nat Ment Health. 2025 Oct 30;3(11):1417–24. doi: 10.1038/s44220-025-00528-x (PMC12589115; doi:10.1038/s44220-025-00528-x)
Supplement: Supplementary file 2 — Reporting Summary [file 44220_2025_528_MOESM2_ESM.pdf]

nature portfolio

Corresponding author(s): Giulia Piazza

Last updated by author(s): Aug 13, 2025

Reporting Summary

Nature Portfolio wishes to improve the reproducibility of the work that we publish. This form provides structure for consistency and transparency in reporting. For further information on Nature Portfolio policies, see our Editorial Policies and the Editorial Policy Checklist.

Statistics

For all statistical analyses, confirm that the following items are present in the figure legend, table legend, main text, or Methods section.

n/a	Confirmed	
☐	☒	The exact sample size (*n*) for each experimental group/condition, given as a discrete number and unit of measurement
☐	☒	A statement on whether measurements were taken from distinct samples or whether the same sample was measured repeatedly
☐	☒	The statistical test(s) used AND whether they are one- or two-sided<br>*Only common tests should be described solely by name; describe more complex techniques in the Methods section.*
☐	☒	A description of all covariates tested
☐	☒	A description of any assumptions or corrections, such as tests of normality and adjustment for multiple comparisons
☐	☒	A full description of the statistical parameters including central tendency (e.g. means) or other basic estimates (e.g. regression coefficient) AND variation (e.g. standard deviation) or associated estimates of uncertainty (e.g. confidence intervals)
☐	☒	For null hypothesis testing, the test statistic (e.g. *F*, *t*, *r*) with confidence intervals, effect sizes, degrees of freedom and *P* value noted<br>*Give P values as exact values whenever suitable.*
☒	☐	For Bayesian analysis, information on the choice of priors and Markov chain Monte Carlo settings
☒	☐	For hierarchical and complex designs, identification of the appropriate level for tests and full reporting of outcomes
☐	☒	Estimates of effect sizes (e.g. Cohen's *d*, Pearson's *r*), indicating how they were calculated

Our web collection on statistics for biologists contains articles on many of the points above.

Software and code

Policy information about availability of computer code

Data collection	This is a secondary analysis of existing data - no software or code was developed to collect data for this study.
Data analysis	The data was analysed using R version 4.2.0. The code is available at https://github.com/giuliapiazza18/PANDAnet-2/

For manuscripts utilizing custom algorithms or software that are central to the research but not yet described in published literature, software must be made available to editors and reviewers. We strongly encourage code deposition in a community repository (e.g. GitHub). See the Nature Portfolio guidelines for submitting code & software for further information.

Data

Policy information about availability of data

All manuscripts must include a data availability statement. This statement should provide the following information, where applicable:

- Accession codes, unique identifiers, or web links for publicly available datasets
- A description of any restrictions on data availability
- For clinical datasets or third party data, please ensure that the statement adheres to our policy

All de-identified individual participant data collected in the PANDA trial, and related documents (study protocol, analysis plan and code) is available, with no end, from the publications of the original trial paper. To gain access, researchers will need to enter a data access agreement with University College London (London, UK), providing a proposal for the use of data and a request for access (glyn.lewis@ucl.ac.uk).

Research involving human participants, their data, or biological material

Policy information about studies with human participants or human data. See also policy information about sex, gender (identity/presentation), and sexual orientation and race, ethnicity and racism.

Reporting on sex and gender	We use the term sex, as provided by the PANDA study, as reported in Lewis et al., 2019, The Lancet, 10.1016/S2215-0366(19)30366-9. The percentage of female participants in treatment and placebo groups is presented in the manuscript.
Reporting on race, ethnicity, or other socially relevant groupings	We use the terms provided by the PANDA study on ethnicity, as reported in Lewis et al., 2019, The Lancet, 10.1016/S2215-0366(19)30366-9
Population characteristics	The PANDA trial recruited 653 patients aged 18-74 across surgery sites in Bristol, York, London and Liverpool who had depressive symptoms in the part 2 years, as reported in Lewis et al., 2019, The Lancet, 10.1016/S2215-0366(19)30366-9
Recruitment	Participants of the PANDA trial were either referred during GP consultation or identified and contacted through searches of computerised records by GP surgeries.
Ethics oversight	Ethics approval was obtained from the National Research Ethics Service committee, East of England—Cambridge South (ref 13/EE/0418).

Note that full information on the approval of the study protocol must also be provided in the manuscript.

Field-specific reporting

Please select the one below that is the best fit for your research. If you are not sure, read the appropriate sections before making your selection.

☐ Life sciences ☒ Behavioural & social sciences ☐ Ecological, evolutionary & environmental sciences

For a reference copy of the document with all sections, see nature.com/documents/nr-reporting-summary-flat.pdf

Behavioural & social sciences study design

All studies must disclose on these points even when the disclosure is negative.

Study description	This study is a quantitative secondary analysis of data from the PANDA trial, a randomised double-blind placebo-controlled randomised trial on the effectiveness of sertraline in primary care in the UK.
Research sample	The sample was obtained from the PANDA trial, which included male and female patients aged 18 to 74 years who had depressive symptoms of any severity or duration in the past 2 years, and reflected the current use of antidepressants in primary care in the UK.
Sampling strategy	The PANDA trial recruited patients from GP surgeries in London, York, Bristol and Liverpool, either through referral during GP consultation or identification through search of computerised GP records. The full details on recruitment, treatment allocation and randomisation are described in the primary PANDA paper and in the trial protocol (referenced in the manuscript). In brief, the sample size was obtained based on power calculations reported in the trial protocol, assuming 90% power, two-sided alpha = 5%, and 10% attrition, for an 11% relative difference in PHQ-9 scores between sertraline and placebo.
Data collection	The PANDA team assessed patient's depression and anxiety symptoms with the Patient Health Questionnaire, 9-item version (PHQ-9) the Beck Depression Inventory (BDI-II), and the Generalised Anxiety Disorder Assessment 7-item version (GAD-7). Physical and mental health measured were assessed with the Short-Form Health Survey (SF-12). Researchers were blind to the randomisation. Further details can be found in Lewis et al., 2019, The Lancet, 10.1016/S2215-0366(19)30366-9
Timing	1 January 2015 - 31 August 2017
Data exclusions	No data was excluded in these analyses
Non-participation	All participants are included in these analyses
Randomization	Particiants were randomly assigned (1:1) to placebo or sertraline with a computer- generated code by PRIMENT Clinical Trials Unit (CTU), stratified by severity, duration and site with random block.

Reporting for specific materials, systems and methods

We require information from authors about some types of materials, experimental systems and methods used in many studies. Here, indicate whether each material, system or method listed is relevant to your study. If you are not sure if a list item applies to your research, read the appropriate section before selecting a response.

Materials & experimental systems

n/a	Involved in the study
☒	☐ Antibodies
☒	☐ Eukaryotic cell lines
☒	☐ Palaeontology and archaeology
☒	☐ Animals and other organisms
☐	☒ Clinical data
☒	☐ Dual use research of concern
☒	☐ Plants

Methods

n/a	Involved in the study
☒	☐ ChIP-seq
☒	☐ Flow cytometry
☒	☐ MRI-based neuroimaging

Clinical data

Policy information about clinical studies

All manuscripts should comply with the ICMJE guidelines for publication of clinical research and a completed CONSORT checklist must be included with all submissions.

Clinical trial registration: ISRCTN84544741

Study protocol: https://pubmed.ncbi.nlm.nih.gov/29065916/

Data collection: Participants were recruited from 179 primary care practices in four UK sites (Bristol, Liverpool, London, York) between Jan 1, 2015, and Aug 31, 2017.

Outcomes: The primary outcome of the PANDA trial was depressive symptoms 6 weeks after randomisation, measured by Patient Health Questionnaire, 9-item version (PHQ-9) scores. Secondary outcomes at 2, 6 and 12 weeks were depressive symptoms and remission (PHQ-9 and Beck Depression Inventory-II), generalised anxiety symptoms (Generalised Anxiety Disorder Assessment 7-item version), mental and physical health-related quality of life (12-item Short-Form Health Survey), and self-reported improvement. Questionnaire data (PHQ-9, BDI-II, GAD-7, SF-12) was used in the secondary analysis of the PANDA trial described in this study.

Plants

Seed stocks: *Report on the source of all seed stocks or other plant material used. If applicable, state the seed stock centre and catalogue number. If plant specimens were collected from the field, describe the collection location, date and sampling procedures.*

Novel plant genotypes: *Describe the methods by which all novel plant genotypes were produced. This includes those generated by transgenic approaches, gene editing, chemical/radiation-based mutagenesis and hybridization. For transgenic lines, describe the transformation method, the number of independent lines analyzed and the generation upon which experiments were performed. For gene-edited lines, describe the editor used, the endogenous sequence targeted for editing, the targeting guide RNA sequence (if applicable) and how the editor was applied.*

Authentication: *Describe any authentication procedures for each seed stock used or novel genotype generated. Describe any experiments used to assess the effect of a mutation and, where applicable, how potential secondary effects (e.g. second site T-DNA insertions, mosiacism, off-target gene editing) were examined.*
